# Bridging ecological processes to elevated antibiotic resistance risk in tomato microbiome under fungicide stress

**DOI:** 10.1093/ismejo/wrag182

**Published:** 2026-07-12

**Authors:** Conglai Zheng, Jiajin Song, Mei Shan, Houpu Zhang, Mengting Qiu, Luqing Zhang, Yunlong Yu, Xiuguo Wang, Hua Fang

**Affiliations:** Zhejiang Key Laboratory of Biology and Ecological Regulation of Crop Pathogens and Insects, Ministry of Agriculture and Rural Affairs Key Laboratory and Molecular Biology of Crop Pathogens and Insect Pests, Zhejiang Engineering Research Center for Biological Control of Crop Pathogens and Insect Pests, Institute of Pesticide and Environmental Toxicology, College of Agriculture and Biotechnology, Zhejiang University, Hangzhou 310058, China; Zhejiang Key Laboratory of Biology and Ecological Regulation of Crop Pathogens and Insects, Ministry of Agriculture and Rural Affairs Key Laboratory and Molecular Biology of Crop Pathogens and Insect Pests, Zhejiang Engineering Research Center for Biological Control of Crop Pathogens and Insect Pests, Institute of Pesticide and Environmental Toxicology, College of Agriculture and Biotechnology, Zhejiang University, Hangzhou 310058, China; Zhejiang Key Laboratory of Biology and Ecological Regulation of Crop Pathogens and Insects, Ministry of Agriculture and Rural Affairs Key Laboratory and Molecular Biology of Crop Pathogens and Insect Pests, Zhejiang Engineering Research Center for Biological Control of Crop Pathogens and Insect Pests, Institute of Pesticide and Environmental Toxicology, College of Agriculture and Biotechnology, Zhejiang University, Hangzhou 310058, China; Anhui Provincial Key Laboratory of Hazardous Factors and Risk Control of Agri-food Quality Safety, College of Resources and Environment, Anhui Agricultural University, Hefei 230036, China; Zhejiang Key Laboratory of Biology and Ecological Regulation of Crop Pathogens and Insects, Ministry of Agriculture and Rural Affairs Key Laboratory and Molecular Biology of Crop Pathogens and Insect Pests, Zhejiang Engineering Research Center for Biological Control of Crop Pathogens and Insect Pests, Institute of Pesticide and Environmental Toxicology, College of Agriculture and Biotechnology, Zhejiang University, Hangzhou 310058, China; Zhejiang Key Laboratory of Biology and Ecological Regulation of Crop Pathogens and Insects, Ministry of Agriculture and Rural Affairs Key Laboratory and Molecular Biology of Crop Pathogens and Insect Pests, Zhejiang Engineering Research Center for Biological Control of Crop Pathogens and Insect Pests, Institute of Pesticide and Environmental Toxicology, College of Agriculture and Biotechnology, Zhejiang University, Hangzhou 310058, China; Zhejiang Key Laboratory of Biology and Ecological Regulation of Crop Pathogens and Insects, Ministry of Agriculture and Rural Affairs Key Laboratory and Molecular Biology of Crop Pathogens and Insect Pests, Zhejiang Engineering Research Center for Biological Control of Crop Pathogens and Insect Pests, Institute of Pesticide and Environmental Toxicology, College of Agriculture and Biotechnology, Zhejiang University, Hangzhou 310058, China; Tobacco Research Institute, Chinese Academy of Agricultural Sciences (CAAS), Qingdao 266101, China; Zhejiang Key Laboratory of Biology and Ecological Regulation of Crop Pathogens and Insects, Ministry of Agriculture and Rural Affairs Key Laboratory and Molecular Biology of Crop Pathogens and Insect Pests, Zhejiang Engineering Research Center for Biological Control of Crop Pathogens and Insect Pests, Institute of Pesticide and Environmental Toxicology, College of Agriculture and Biotechnology, Zhejiang University, Hangzhou 310058, China

**Keywords:** fungicide, antibiotic resistance genes, plant microbiome, ecological process, ecological risk

## Abstract

From a “One Health” perspective, antibiotic resistance genes (ARGs) harbored by the plant microbiome pose a significant threat to public health, yet their ecological mechanisms under fungicide stress remain largely unexplored. Here, a comprehensive framework integrating selection, dispersal, antagonistic interactions, and horizontal gene transfer (HGT) is established to elucidate the ecological risks and assembly mechanisms of the tomato resistome under fungicide stress, using multi-omics and several validation experiments. The indirect/direct ecological risks of ARGs in aboveground tomato tissues increase by 1.69–93.81-fold and 1.29–123.49-fold under fungicide exposure, respectively, compared to the control. Dispersal and selection emerge as the dominant ecological processes shaping the resistome under fungicide stress, driven by antibiotic-resistant bacteria (ARB) with streamlined and multifunctional metabolic traits, respectively. A fluorescently labeled ARB migration model and an indigenous ARB-based conjugation model demonstrate that fungicides promote the upward dispersal of native ESKAPE pathogens and intensify HGT among them, facilitating the emergence of multidrug-resistant bacteria. Validation experiments confirm that fungicides induce metabolic reprogramming of flavonoid biosynthesis in roots, which enhances HGT by modulating various physiological phenotypes. These findings underscore the ecological risks posed by fungicides in promoting ARG dissemination within the plant microbiome through multiple ecological mechanisms.

## Graphical Abstract

**Graphical Abstract:**
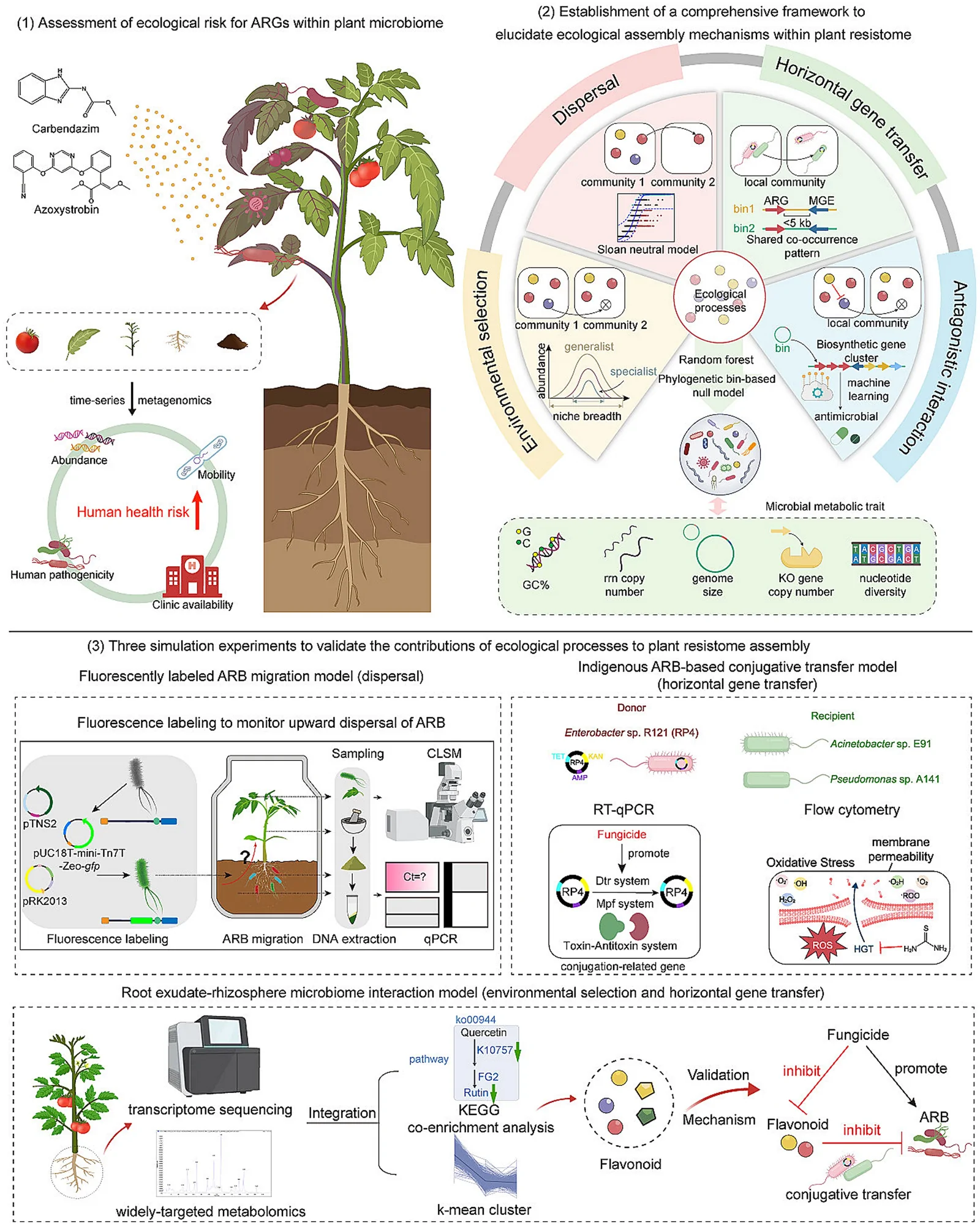

## Introduction

Antibiotic resistance genes (ARGs), as emerging environmental contaminants, have been recognized as a major global threat to human health in the 21st century [1]. From a “One Health” perspective, the agroecosystems, including animals, plants, water, air, and soil, constitute interconnected microbial habitats that serve as a major reservoir of clinically relevant ARGs [2]. Within this continuum, the plant microbiome functions as a critical interface connecting natural and human-associated microbiome through the food chain [3]. Given the escalating concern over antibiotic resistance, assessing the evolution, drivers, and potential health risks of ARGs within the plant microbiome is essential for informing strategies to curb the spread of resistance in agroecosystems.

Accumulating evidence indicates that non-antibiotic substances such as heavy metals, disinfectants, and pesticides are important drivers of antibiotic resistance in natural environments [4]. Pesticides are crucial to modern agriculture for protecting crops from pathogens and pests. Climate change and agricultural intensification-induced crop losses (17%–30%) due to uncontrolled pests, pathogens, and weeds have driven a 6-fold increase in global agricultural pesticide usage from 1950s to 2021 [5]. Several studies have shown that certain fungicides exert dual effects on ARG transfer: they not only impose selective pressure that enriches antibiotic-resistant bacteria (ARB) but also enhance horizontal gene transfer (HGT) [6]. Despite these advances, how the plant resistome responds to fungicide stress remains poorly understood, particularly under field conditions.

Deciphering the ecological processes that govern microbial community assembly is central to microbial ecology and critical for understanding the evolution of the antibiotic resistome [7]. The plant microbiome is shaped by diverse biotic and abiotic factors, including host genotype, developmental stage, soil properties, insects, and environmental fluctuations [8]. These drivers are generally quantified into deterministic (e.g., environmental selection and biotic interactions) and stochastic (e.g., dispersal and genetic mutation) ecological processes [9]. The plant-associated microbiome occupies distinct niches, including root, stem, leaf, and fruit, each defined by unique environmental conditions and plant–microbe crosstalk [10]. For instance, the rhizosphere forms a nutrient-enriched hotspot shaped by root exudates, whereas the phyllosphere constitutes an open habitat exposed to nutrient scarcity and fluctuating abiotic stress [10]. Yet how such spatial heterogeneity modulates the assembly of the plant microbiome and resistome under environmental stress remains unclear. Ecological processes are strongly regulated by microbial genetic diversity and life-history strategies, including growth, competition, stress tolerance, resource acquisition, and dispersal capacity [11]. However, the mechanisms by which metabolic traits mediate resistome assembly under fungicide stress remain largely unexplored.

**Figure 1:**
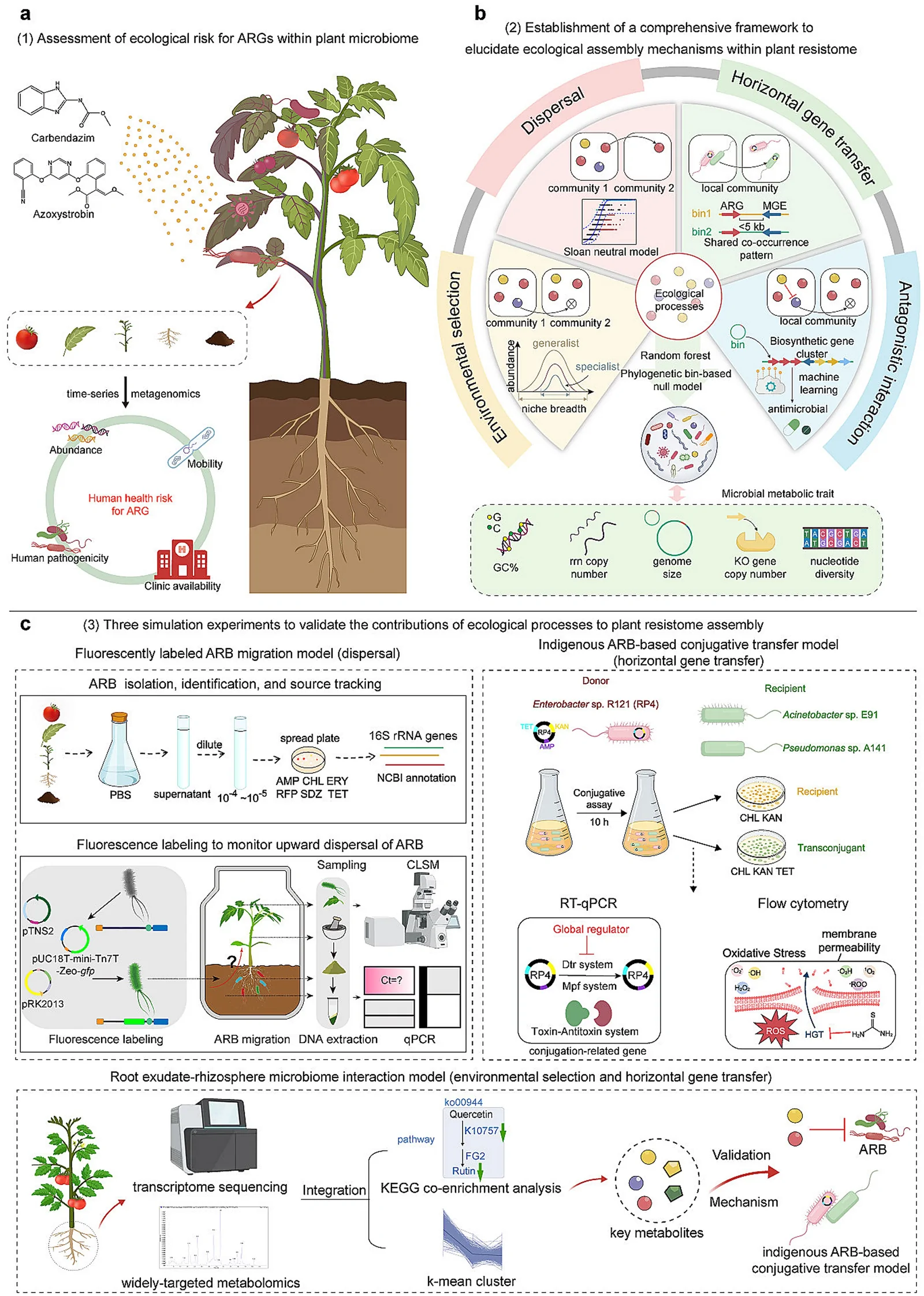
Schematic diagram of the study. (**a**) The ecological risks of the tomato resistome under fungicide (carbendazim and azoxystrobin) stress were assessed by integrating the abundance, mobility, human pathogenicity, and clinical availability of ARGs based on 180 time-series metagenomes obtained from laboratory and field tomato cultivation systems (encompassing fruit, leaf, stem, root, and soil). (**b**) A comprehensive framework integrating environmental selection, dispersal, HGT, and antagonistic interactions was established to elucidate the ecological assembly mechanisms of the tomato resistome. The role of microbial metabolic traits in mediating resistome assembly was determined by linking key ecological processes with microbial life-history strategies, genetic diversity, and KEGG functional profiles. (**c**) Three validation experiments were conducted to disentangle the contributions of different ecological processes (dispersal, HGT, and environmental selection) to resistome assembly: (i) fluorescently labeled antibiotic-resistant bacteria (ARB) migration model; (ii) indigenous ARB-based conjugative transfer model; and (iii) root exudate-rhizosphere microbiome interaction model. AMP: ampicillin; CHL: chloramphenicol; ERY: erythromycin; RFP: rifampicin; SDZ: sulfadiazine; TET: tetracycline; KAN: kanamycin; CLSM: confocal laser scanning microscopy.

In this study, a comprehensive framework was established to reveal the ecological assembly mechanisms of the plant resistome under the stress of two representative fungicides, azoxystrobin (AZO) and carbendazim (CBD), by 180 time-series metagenomes from laboratory/field tomato cultivation systems (Fig. 1). The relative contributions of dispersal, selection, antagonistic interactions, and HGT, as well as their interactions with microbial metabolic traits, were quantified by integrating neutral theory, niche theory, random forest model, and phylogenetic bin-based null model [9]. To experimentally validate the contributions of dispersal, HGT, and selection to resistome assembly, we further constructed: (i) a fluorescently labeled ARB migration model, (ii) an indigenous ARB-based conjugative transfer model, and (iii) a root exudate-rhizosphere microbiome interaction model. The framework herein underscores the pivotal role of fungicides in shaping the ecological risk of the plant resistome and provides insights into the ecological mechanism of plant resistome development.

## Materials and methods

### Chemicals and reagents

Chemicals and reagents are detailed in Supplementary Method 1.

### Laboratory pot experiment and greenhouse experiment

To investigate the effects of carbendazim and azoxystrobin on the resistome in the soil–plant system (roots, stems, leaves, and fruits), laboratory pot experiments and greenhouse experiments were conducted and analyzed separately using cherry tomato (*Solanum lycopersicum var. cerasiforme*), as detailed in Supplementary Methods 2–4. The results of laboratory pot experiments are only described in Supplementary Results 1–2.

### Extraction and determination of fungicide residues

The methods for extraction and quantification of fungicide residues are detailed in Supplementary Methods 5–9. Residue dynamics of carbendazim and azoxystrobin in both laboratory and field-based tomato cultivation systems are described in Supplementary Result 2.

### DNA extraction and metagenomic sequencing

Tomato root, stem, leaf, and fruit samples were pretreated to reduce host DNA contamination, and total DNA was then extracted and sent for metagenomic sequencing, which is detailed in Supplementary Method 10.

### Resistome and microbiome analyses

ARGs were identified by aligning metagenomic sequences against the Comprehensive Antibiotic Resistance Database (CARD) using BLASTX [12]. Hits were filtered based on the following criteria: amino acid identity ≥90%, alignment length ≥ 25 amino acids, and E-value <1e–5. ARG abundances were normalized to bacterial cell numbers (calculated as the mean copy number of essential bacterial single-copy marker genes) and expressed as “copies per cell” [13]. Overall differences in ARG profiles were assessed using ANOSIM, and SIMPER analysis was subsequently performed to identify indicator ARGs. The absolute abundance of six dominant ARGs in the tomato cultivation system was further quantified using qPCR, as detailed in Supplementary Method 11. The methods for microbiome analysis are detailed in Supplementary Method 12.

### Human health risk of the resistome

Metagenomic contigs (≥500 bp) were assembled using metaSPAdes [14]. Open reading frames (ORFs) were predicted from contigs using Prodigal, and ORFs were aligned against the Comprehensive Antibiotic Resistance Database (CARD) [15] using DIAMOND [16] and BLASTP with thresholds of ≥90% sequence identity and ≥70% coverage to identify ARGs. To identify mobile genetic elements (MGEs) and virulence factor genes (VFGs), ORFs were further aligned against the mobileOG [17] and VFDB [18] databases using DIAMOND and BLASTP with thresholds of ≥90% identity and ≥90% coverage. The abundance of MGEs and VFGs was calculated by mapping reads back to contigs using BWA [19]. To investigate potential mobility and pathogenicity of ARGs, co-occurrence patterns of ARGs, MGEs, and VFGs on contigs were analyzed. Contigs carrying both ARGs and VFGs were classified as direct risk, whereas contigs harboring both ARGs and MGEs, but lacking VFGs, were classified as indirect risk [20]. The relative abundance of direct and indirect risk contigs was normalized to coverage/Gb using CheckM [21]. The human health risk (HHR) index for each ARG subtype was calculated using the following formula:


$$ {HHR}_i=\frac{\sum_1^n{A}_i\times \frac{A_M}{A_{contig}}+{A}_i\times \frac{A_P}{A_{contig}}}{n} $$

A_i_ is the normalized abundance of ARG_i_ (coverage/Gb), A_contig_ is the abundance of contigs carrying ARG_i_, A_M_ is the abundance of contigs carrying both ARG_i_ and MGEs but not VFGs (indirect risk), A_P_ is the abundance of contigs carrying both ARG_i_ and VFGs (direct risk), and n is the number of samples.

Based on the HHR index, ARGs were ranked into high-priority (top 0%–33%), medium-priority (33%–67%), and low-priority (67%–100%). Additionally, ARGs were prioritized by the clinical availability of antimicrobial classes to which they confer resistance according to the World Health Organization’s list of critically important antimicrobials [22]. ARGs with high clinical importance or HHR were designated as highest-priority ARGs. To further assess HGT potential, clean reads were assembled to plasmids using metaplasmidSPAdes [23], and mobilizable/conjugative plasmids were identified using Plascad [24].

### Ecological assembly modeling of the resistome

Metagenome-assembled genomes (MAGs) were reconstructed using MetaWRAP [25]. Then bins were refined using the bin_refinement and bin_reassembly modules to extract high-quality genomes. Only MAGs with ≥70% completeness and ≤10% contamination were retained. Redundancy among MAGs was removed using dRep [26]. Taxonomic classification and quantification of MAGs were performed using the classify_wf module of GTDB-Tk [27] (GTDB Release R220) and the quant_bins module of MetaWRAP, respectively. Phylogenetic trees were constructed using IQ-TREE2 [28]. A strict workflow was developed to detect potential HGT events, requiring the following criteria to be met: (i) Co-localization of ARGs with MGEs (eg, plasmids, transposons, or insertion sequences); (ii) Identification of the shared ARG–MGE co-occurrence pattern in MAGs from distinct species (average nucleotide identity (ANI) <95%); and (iii) Presence of ancestral MAGs (ANI > 95%) without ARGs.

Based on niche theory, the niche breadth of ARG-carrying MAGs was quantified using the R package “spaa” [29] to characterize the strength of selection. Dispersal of ARG-carrying MAGs was calculated as the dispersal parameter (m) of the Sloan neutral model, via the R packages “Hmisc”, “minpack.lm”, and “stats4” [30]. Biosynthetic gene clusters (BGCs) in MAGs were predicted using antiSMASH [31], and machine learning models were employed to predict the antimicrobial activity of BGCs (aBGCs) [32]. The relative abundance of MAGs harboring aBGCs was used to characterize antagonistic interactions [33]. HGT potential was estimated based on the relative abundance of ARG-carrying MGEs. The relative importance of ecological processes, including selection, dispersal, antagonistic interaction, and HGT, in shaping the resistome was assessed using random forest models by the R packages “randomForest” and “rfPermute”, and model significance of random forest was evaluated using the “A3” package [34]. Furthermore, phylogenetic bin-based null model was conducted by the R package “iCAMP” [9]. Source tracking of ARB and their upward migration potential was quantified using the R package “FEAST” [35].

### Functional annotation, life-history strategy, and genetic diversity

Functional annotation of ORFs was performed using eggNOG-mapper v2 [36] by aligning sequences against the eggNOG database. The relative abundance of functional genes was quantified using BWA [19]. The contribution of individual KEGG pathways to functional β-diversity was assessed via SIMPER analysis using the R package “vegan” [37]. To infer microbial life-history strategies, ribosomal RNA operons (*rrn*) copy number and average genome size (AGS) were estimated using ACN [38] and MicrobeCensus [39], respectively. GC content was calculated using FastQC [40], and the ratio of copiotrophic to oligotrophic taxa was estimated following a previous study [41]. These four traits of *rrn* copy number, AGS, GC content, and copiotroph/oligotroph ratio were integrated using principal component analysis (PCA), and the first principal component (PCA1) was used to represent the r-/K- strategy ratio. Genetic diversity was assessed using -profile workflow of InStrain [42], including nucleotide diversity, nonsynonymous to synonymous substitution ratios (dN/dS), and linkage disequilibrium (D′).

### Isolation of antibiotic-resistant bacteria

Isolation of ARB from the greenhouse experiments and antibiotic susceptibility testing are detailed in Supplementary Methods 13, 14.

### Construction of fluorescently labeled ARB migration model

Seven representative ARB were selected, including *Acinetobacter* sp. E91, *Klebsiella* sp. S21, *Pseudomonas* sp. A141, *Atlantibacter* sp. A31, *Enterobacter* sp. R121, *Pantoea* sp. A32, and *Achromobacter* sp. T121. A Tn7-based site-specific chromosomal integration system was used to insert the green fluorescent protein (*gfp*) gene downstream of the conserved *glmS* gene in each ARB, generating fluorescently labeled ARB. The integration was achieved via electroporation or quad-parental mating, which was detailed in Supplementary Methods 15–19. To investigate ARB migration from soil to plant tissues under fungicide stress, a fluorescently labeled ARB migration model was established, which is detailed in Supplementary Method 20. The spatial distribution of fluorescently labeled ARB in plant tissues was visualized using confocal laser scanning microscopy (CLSM) [43]. To quantify bacterial colonization in soil and plant tissues, absolute abundance of the *gfp* gene was quantified using qPCR [44]. Visualization and quantification of fluorescently labeled ARB are detailed in Supplementary Methods 21, 22.

### Construction of an indigenous ARB-based conjugative transfer model

Detailed protocols for plasmid curing, antibiotic susceptibility testing, and donor strain construction are provided in Supplementary Methods 23, 24. Based on the MIC results for representative indigenous strains, we selected *Enterobacter* sp. R121(RP4) as donor strains, and *Acinetobacter* sp. E91 and *Pseudomonas* sp. A141 as recipient strains. An indigenous conjugative transfer system was then established, which is described in the Supplementary Methods 25–29. Intracellular reactive oxygen species (ROS) production and membrane permeability of donor and recipient strains were quantified using flow cytometry, as described in Supplementary Methods 30, 31. To investigate whether oxidative stress mediates fungicide-induced horizontal transfer of ARGs, thiourea (100 μM) was used as a ROS scavenger [45], and conjugation frequencies were reassessed under carbendazim and azoxystrobin treatments at 1 and 5 mg/L. To characterize molecular responses to fungicide stress during conjugative transfer, RT-qPCR was used to quantify six key functional genes of plasmids, which is detailed in Supplementary Method 32.

### Construction of a root exudate-rhizosphere microbiome interaction model

Tomato seedlings were cultivated following the protocol described in Supplementary Method 3. A pot-based tomato cultivation system was established by three treatments of control, 5 mg/kg carbendazim, and 5 mg/kg azoxystrobin, with three biological replicates in each treatment. After 14 d, rhizosphere soil, root tissues, and root exudates were collected for RNA extraction, transcriptome sequencing, and widely-targeted metabolomics, which are detailed in Supplementary Methods 33–36. Based on their fold changes and interactions with ARB under fungicide stress, representative differentially accumulated metabolites (DAMs) were selected for mechanistic validation using indigenous antibiotic-resistant strains isolated from the tomato cultivation system. Their antimicrobial activity and effects on ROS production, cell membrane permeability, and plasmid conjugation transfer frequency were determined according to Supplementary Methods 14, 25–31. To evaluate the potential of specific root exudates to mitigate ARG contamination, selected DAMs were applied to fungicide-treated tomato systems according to Supplementary Method 3. Rhizosphere soil and root samples were collected for metagenomic sequencing, and the effect of DAMs on ARG reduction was assessed.

### Statistical analysis and visualization

All data are presented as means ± standard deviations and were analyzed using the “agricolae” package in R [46]. For pairwise comparisons, statistical significance was assessed using either Student’s *t-*test or the Wilcoxon signed-rank test. For multiple treatment comparisons, one-way analysis of variance (ANOVA) was conducted followed by Tukey’s honestly significant difference (HSD) post hoc test. Ordinary least squares (OLS) regression models were used to explore linear relationships between variables. Data visualization was performed using Origin 2020 and R packages including “ggplot2”, “pheatmap”, “ggradar”, and “ggtreeExtra” [47]. Networks were visualized using Gephi [48] and Cytoscape [49]. Other supplementary data, including Supplementary Figs 19–39 and Supplementary Tables 8–19, are provided in the supplementary materials.

## Results

### Ecological risks of the plant resistome under fungicide stress

**Figure 2:**
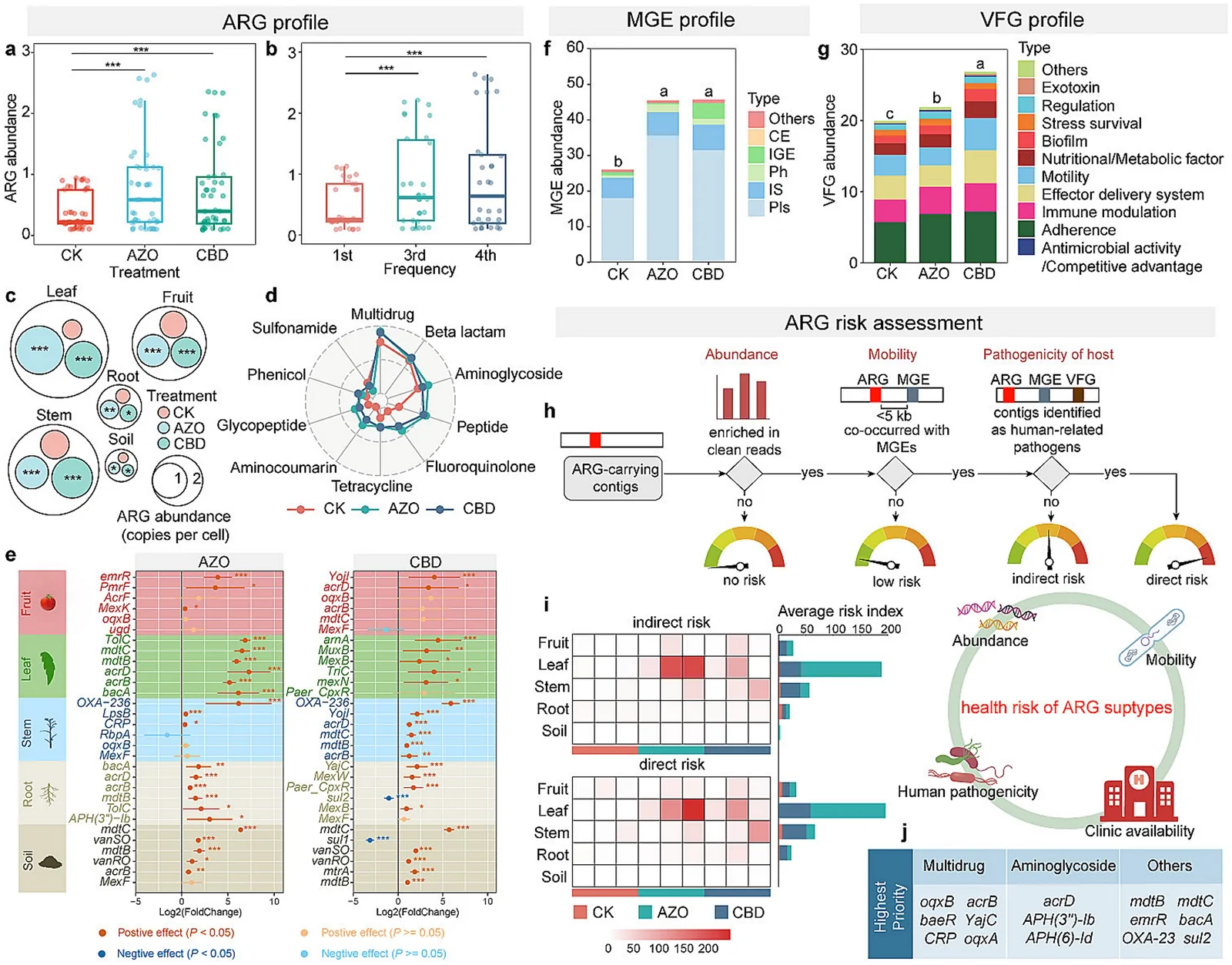
Effects of fungicides on the ecological risk of the antibiotic resistome in tomato microbiome. (**a**, **b**) Effect of fungicide application (**a**) and treatment frequency (**b**) on ARG abundance (Wilcoxon signed-rank test, significance: ^***^ for *P* < .001). (**c**) Effect of fungicide on ARG abundance in different plant tissues and soil (Wilcoxon signed-rank test, significance: ^*^ for *P* < .05, ^**^ for *P* < .01, ^***^ for *P* < .001). (**d**) Effect of fungicide on dominant ARG types. (**e)** Representative ARG subtypes in different plant tissues and soil (Wilcoxon signed-rank test, significance: ^*^ for *P* < .05, ^**^ for *P* < .01, ^***^ for *P* < .001). (**f**, **g**) Effect of fungicide on MGE (**f**) and VFG (**g**) profiles (Pls: Plasmid, IS: Insertion sequence, Ph: Phage, IGE: Integrative element, CE: Conjugative element). (**h**) Pipeline for indirect/direct ecological risk detection. (**i**) Indirect/direct risk indices of ARGs. (**j)** ARG subtypes of highest priority in human health risk. CK: control; AZO: azoxystrobin; CBD: carbendazim; MGE: mobile genetic element; VFG: virulence factor gene.

In laboratory pot experiments, we confirmed the promotive effect of fungicides on the proliferation of ARGs (Supplementary Result 1). Field trials further demonstrated that the total abundance of ARGs within the soil–plant continuum significantly increased with both the application and frequency of fungicide treatments (Wilcoxon signed-rank test, *P* < .001), particularly those conferring resistance to multidrug, aminoglycoside, fluoroquinolone, and tetracycline antibiotics (Fig. 2a, b, d). Spatial heterogeneity of ARG distribution was observed across different plant tissues and soil (Supplementary Fig. 1). More precisely, aboveground tissues exhibited a more pronounced increase in ARG abundance compared to root and soil, with 1.16–2.03-fold increases in stem, 3.15–6.01-fold in leaf, and 1.27–1.44-fold in fruit (Fig. 2c; Wilcoxon signed-rank test, *P* < .001). SIMPER analysis further identified representative ARG subtypes contributing to spatial variation: multidrug (*acrB*, *oqxB*, and *MexF*) and aminocoumarin (*mdtB* and *mdtC*) resistance genes were enriched across all plant tissues, whereas glycopeptide (*vanRO* and *vanSO*) resistance genes were predominantly enriched in root and soil, as validated by quantitative PCR (qPCR; Fig. 2e and Supplementary Fig. 2).

To assess mobility and host pathogenicity of ARGs, we identified six types of MGEs, encompassing 43401 subtypes, and 14 types of VFGs, comprising 2204 subtypes (Fig. 2f, g). Fungicide significantly elevated the relative abundance of plasmids, phages, and VFGs associated with adherence, nutritional/metabolic factors, motility, and biofilm formation (Wilcoxon signed-rank test, *P* < .05; Supplementary Figs 3, 4). Integrating abundance, mobility, host pathogenicity, and clinical availability, we quantified indirect/direct risk indices for ARGs across all samples (Fig. 2h and Supplementary Tables 1, 2). Aboveground tissues represented hotspots for ecological risk (Fig. 2i), in which the mean indirect/direct risk indices of ARGs reached 13.09–93.27 and 12.48–97.94 under fungicide stress, respectively, higher than those in controls (0.14–6.99 and 0.09–7.15). These elevated risk indices were primarily driven by multidrug and aminoglycoside resistance genes (Supplementary Table 3). Specifically, aminoglycoside resistance gene *acrD* frequently co-occurred with conjugative elements such as *trbL*, *trbJ*, and *traC*, whereas multidrug resistance genes *oqxAB* and *acrB* were generally co-located on plasmids with VFGs related to nutritional/metabolic factors (*entABCE* and *fepABCDG*) and adherence (*fimDHZ*) (Fig. 2j and Supplementary Fig. 5).

### Ecological mechanisms underlying the assembly of the plant resistome under fungicide stress

Bacterial communities were recognized as the primary drivers of ARG dissemination (Supplementary Fig. 6a; Procrustes: M^2^ = 0.12, *P* < .001). The aboveground plant microbiome exhibited lower γ-diversity and formed a distinct β-diversity assembly pattern compared to the root and soil microbiome (Supplementary Fig. 6b, c). Variance partitioning analysis (VPA) revealed that ARGs in aboveground tissues were primarily affected by generalists with low community resistance (Supplementary Fig. 6d). In contrast, ARG hosts in root and soil were phylogenetically microdiverse, comprising both generalists and specialists.

**Figure 3:**
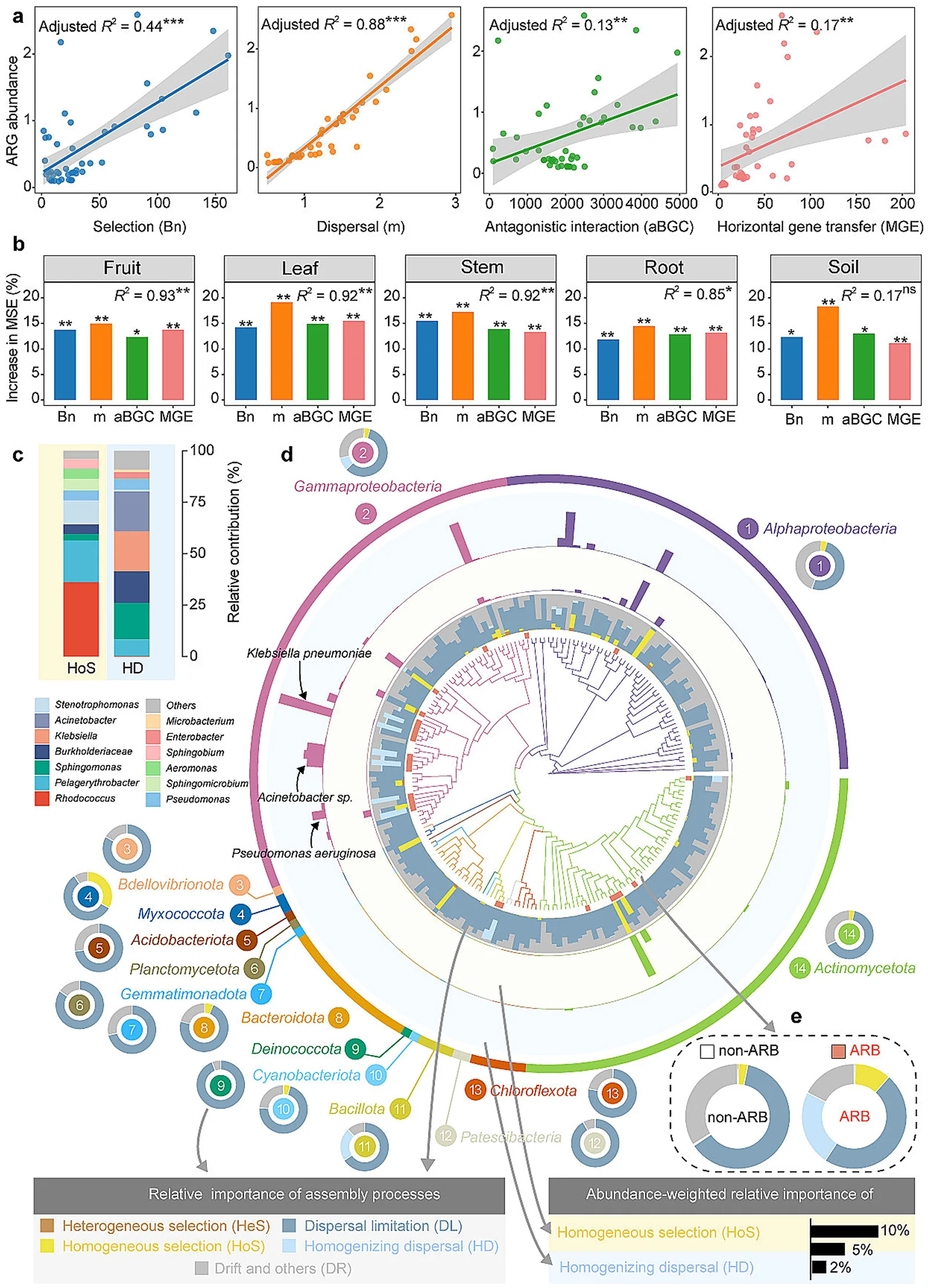
Ecological mechanisms underlying the assembly of the plant resistome under fungicide stress. **(a**) Ordinary least squares (OLS) regression models between the strength of selection, dispersal, antagonistic interaction, HGT and ARG abundance. (**b**) Random forest analysis to predict relative importance of selection, dispersal, antagonistic interaction, and HGT (characterized by Bn, m, aBGC, and MGE, respectively) to ARG abundance in different plant tissues and soil (significance: ^*^ for *P* < .05, ^**^ for *P* < .01). (**c**) Contribution of microbes to ecological processes of homogeneous selection (HoS) and homogeneous dispersal (HD) under fungicide stress. (**d**) Phylogenetic bin-based null model to reveal the ecological mechanisms underlying the assembly of each bacteria under fungicide stress, as well as relative importance of different ecological processes in each phylum (*Proteobacteria* were divided into classes). The contributions of each bin to HoS and HD were quantified under consideration of the abundance of bins. (**e**) Relative importance of different processes in antibiotic-resistant bacteria (ARB) and other taxa (non-ARB) under fungicide stress. Bn: niche breadth; m: migration rate; aBGC: antimicrobial biosynthetic gene cluster; MGE: mobile genetic element.

The phylogenetic structure of ecological communities reflects both evolutionary and ecological processes, providing insights into resistome assembly. Selection, antagonistic interactions, dispersal, and HGT were identified as the primary forces shaping resistome structure, and random forest analysis highlighted dispersal as the most significant predictor of ARG abundance (Fig. 3a, b). A phylogenetic bin-based null model further revealed that dispersal limitation (DL; 62.1%) and drift (DR; 33.8%) dominated community assembly, followed by homogeneous selection (HoS; 2.9%) and homogeneous dispersal (HD; 0.8%) (Fig. 3d). Fungicides shifted assembly patterns: ARB became increasingly shaped by HoS and HD, whereas others remained strongly constrained by DL and DR (Fig. 3e). When accounting for bin-level abundance, HD was largely driven by the responses of *Acinetobacter* and *Klebsiella*, whereas HoS was mainly associated with *Rhodococcus* and *Stenotrophomonas* (Fig. 3c). Moreover, the ecological processes governing ARB were strongly modulated by spatial heterogeneity under fungicide stress. For example, the resistome was primarily shaped by HoS in root and soil, whereas both HD and HoS contributed to leaf resistome assembly (Supplementary Fig. 7). These findings underscore the intertwined roles of selection and dispersal in shaping the plant resistome under fungicide stress.

### Ecological processes are correlated with microbial metabolic traits

**Figure 4:**
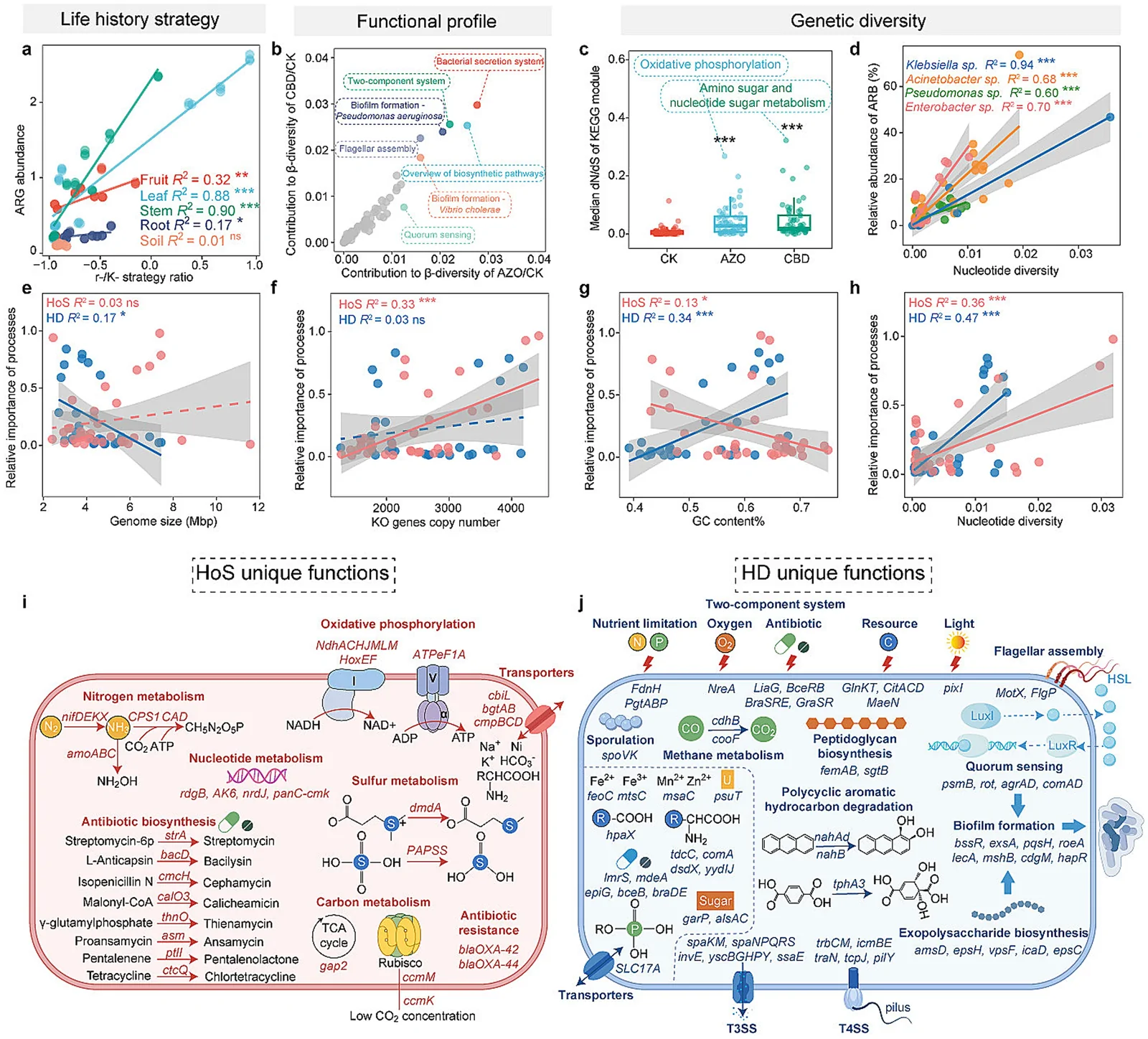
Correlation between ecological processes and microbial metabolic traits. **(a**) Ordinary least squares (OLS) regression models between r-/K- strategy ratio and ARG abundance (significance: ^*^ for *P* < .05, ^**^ for *P* < .01, ^***^ for *P* < .001). (**b**) Contributions of each KEGG ortholog (KO) to functional profile. (**c**) Effect of fungicide on median dN/dS of KEGG modules (Wilcoxon signed-rank test, significance: ^***^ for *P* < .001). (**d**) OLS regression models between nucleotide diversity and relative abundance of antibiotic-resistant bacteria (ARB; significance: ^***^ for *P* < .001). (**e**–**h**) OLS regression models between genome size (**e**), KO genes copy number (**f**), GC content% (**g**), nucleotide diversity (**h**) and relative importance of homogeneous selection (HoS) and homogeneous dispersal (HD; significance: ^*^ for *P* < .05, ^***^ for *P* < .001). (**i**, **j**) Unique KOs in HoS-driven (**i**) and HD-driven (**j**) ARB. CK: control; AZO: azoxystrobin; CBD: carbendazim.

Microbial life-history strategies and genetic diversity determine their metabolic potential and responses to fungicide stress, thereby affecting resistome assembly. Bacterial communities adopted strategies characterized by regulated coding activity, high resource acquisition capacity, rapid growth rates, and streamlined genome sizes under fungicide stress, which collectively favored the enrichment of copiotrophic taxa (Supplementary Fig. 8a and Fig. 4a). Genes associated with biofilm formation, flagellar motility, quorum sensing, and bacterial secretion system promoted the segregation of functional profiles under fungicide stress (Supplementary Fig. 8b and Fig. 4b). Additionally, microbial nucleotide diversity significantly increased following fungicide application, driven by intensified positive selection (higher dN/dS ratios) targeting energy and carbohydrate metabolism pathways rather than homologous recombination (Wilcoxon signed-rank test, *P* < .05; Fig. 4c and Supplementary Fig. 8c, d). The nucleotide diversity of copiotrophic ARB such as *Klebsiella*, *Acinetobacter*, *Pseudomonas*, and *Enterobacter* was positively correlated with their relative abundance in tomato tissues, suggesting that genetic mutations facilitated their colonization capacity (Fig. 4d).

Given the dominance of HoS and HD in ARB assembly under fungicide stress, we investigated how metabolism-related factors contribute to these processes. ARB predominantly exhibited two distinct strategies: (i) a streamlined strategy, characterized by smaller genomes and higher GC content, driven by HD; and (ii) a multifunctional strategy, marked by higher functional diversity and lower GC content, driven by HoS (Fig. 4e-g). Moreover, genetic diversity was positively correlated with both HoS and HD (Fig. 4h).

To further dissect the unique metabolic capabilities of these strategies, we further classified ARB based on the relative importance of HoS and HD and identified their distinct KEGG Orthologs (KO). ARB dominated by HoS (HoS > 30%; HoS > HD), mainly *Mycobacteriaceae*, *Xanthomonadaceae*, *Sphingomonadaceae*, and *Burkholderiaceae*, exhibited enrichment of 208 unique KOs related to specialized metabolic pathway (nutrient cycling and energy metabolism) and competition (antibiotic biosynthesis) (Supplementary Table 4 and Fig. 4i). In contrast, ARB dominated by HD (HD > 30%; HD > HoS), primarily *Enterobacteriaceae*, *Moraxellaceae*, and *Pseudomonadaceae*, enriched 354 unique KOs involved in environmental sensing (two-component systems), adaptation (quorum sensing, exopolysaccharide biosynthesis, and biofilm formation), and motility (flagellar assembly). Two-component system-related KOs were particularly associated with responses to environmental signals such as nutrient limitation, oxygen, antibiotics, resource availability, and light (Supplementary Table 4 and Fig. 4j).

### Fungicide stress promotes the dispersal of antibiotic-resistant pathogens

**Figure 5:**
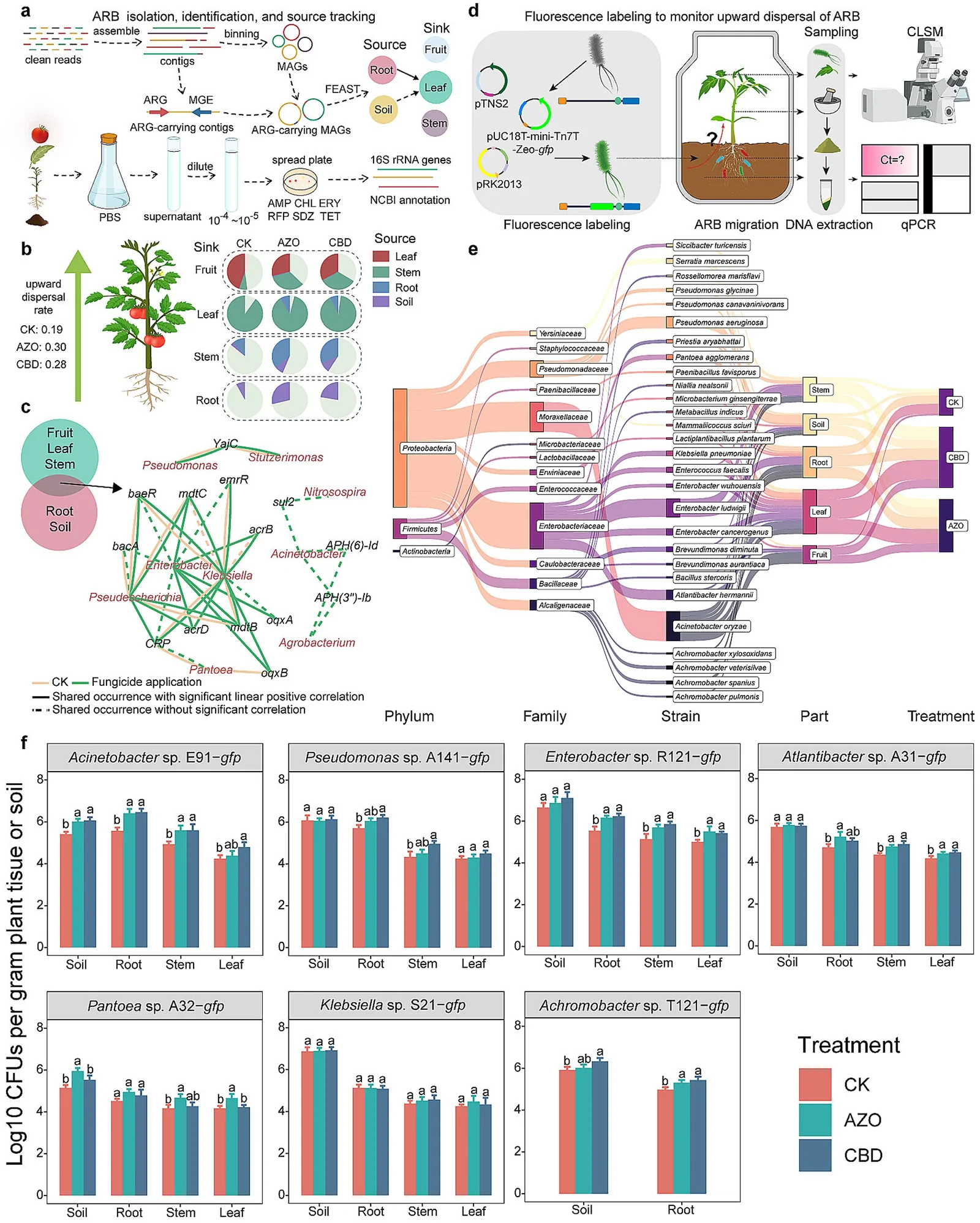
Effect of fungicide stress on the dispersal of antibiotic-resistant bacteria especially for opportunistic pathogens. **(a**) Schematic illustration of antibiotic-resistant bacteria (ARB) isolation, identification, and source tracing. Metagenomic binning combined with fast expectation–maximization for microbial source tracking (FEAST) analysis was first used for source-tracking of ARB, followed by enrichment culturing to isolate ARB from different tomato tissues for experimental validation. (**b**) FEAST source-tracking analysis to characterize microbial upward dispersal rate. (**c**) Shared ARG-ARB co-occurrence with/without significant linear positive correlation in different treatments. (**d**) Schematic diagram of fluorescently labeled ARB migration model. Seven indigenous antibiotic-resistant strains isolated from the tomato cultivation system were fluorescently labeled using a Tn7-based site-specific chromosomal integration system, inserting a *gfp* gene downstream of the conserved *glmS* locus. The migration of fluorescently labeled ARB from soil to plant tissues was visualized and quantified by CLSM and quantitative PCR (qPCR). (**e**) Source-tracking of ARB isolated from various plant tissues and soils using enrichment culturing. (**f**) Quantification of ARB colonization in soil, root, stem, and leaf. CK: control; AZO: azoxystrobin; CBD: carbendazim.

Fast expectation–maximization for microbial source tracking (FEAST) analysis further confirmed the critical role of dispersal in the enrichment of ARGs (Fig. 5a, b). The abundance of 15 highest-priority ARGs was significantly and positively correlated with relative abundances of shared ARB, particularly *Klebsiella* and *Enterobacter*, in both aboveground and belowground tissues under fungicide exposure (Fig. 5c). 10 of 15 highest-priority ARGs were encoded by metagenome-assembled genomes (MAGs) belonging to these genera (Supplementary Fig. 9). In contrast, such correlations and sharing patterns were rarely observed in controls. Consistently, multiple shared antibiotic-resistant ESKAPE pathogens were isolated from plant tissues and soils under fungicide exposure (Fig. 5e and Supplementary Fig. 10). *Acinetobacter* and *Enterobacter* isolates carried diverse multidrug, tetracycline, β-lactam, and MLS resistance genes and exhibited corresponding resistance to ampicillin, erythromycin, and tetracycline (Supplementary Table 5). We then selected seven representative antibiotic-resistant strains to construct a fluorescently labeled ARB migration model (Fig. 5d). CLSM revealed that most ARB were capable of upward migration, although their movement toward stem and leaf was impeded (Supplementary Fig. 11). Fungicides enhanced the internal migration of ESKAPE pathogens like *Enterobacter ludwigii* and *Pseudomonas aeruginosa* from soil into tomato tissues, and their maximum migration quantities exceeded those in control treatments by more than 4-fold (Fig. 5f).

### Fungicides promote the emergence of multidrug-resistant bacteria

**Figure 6:**
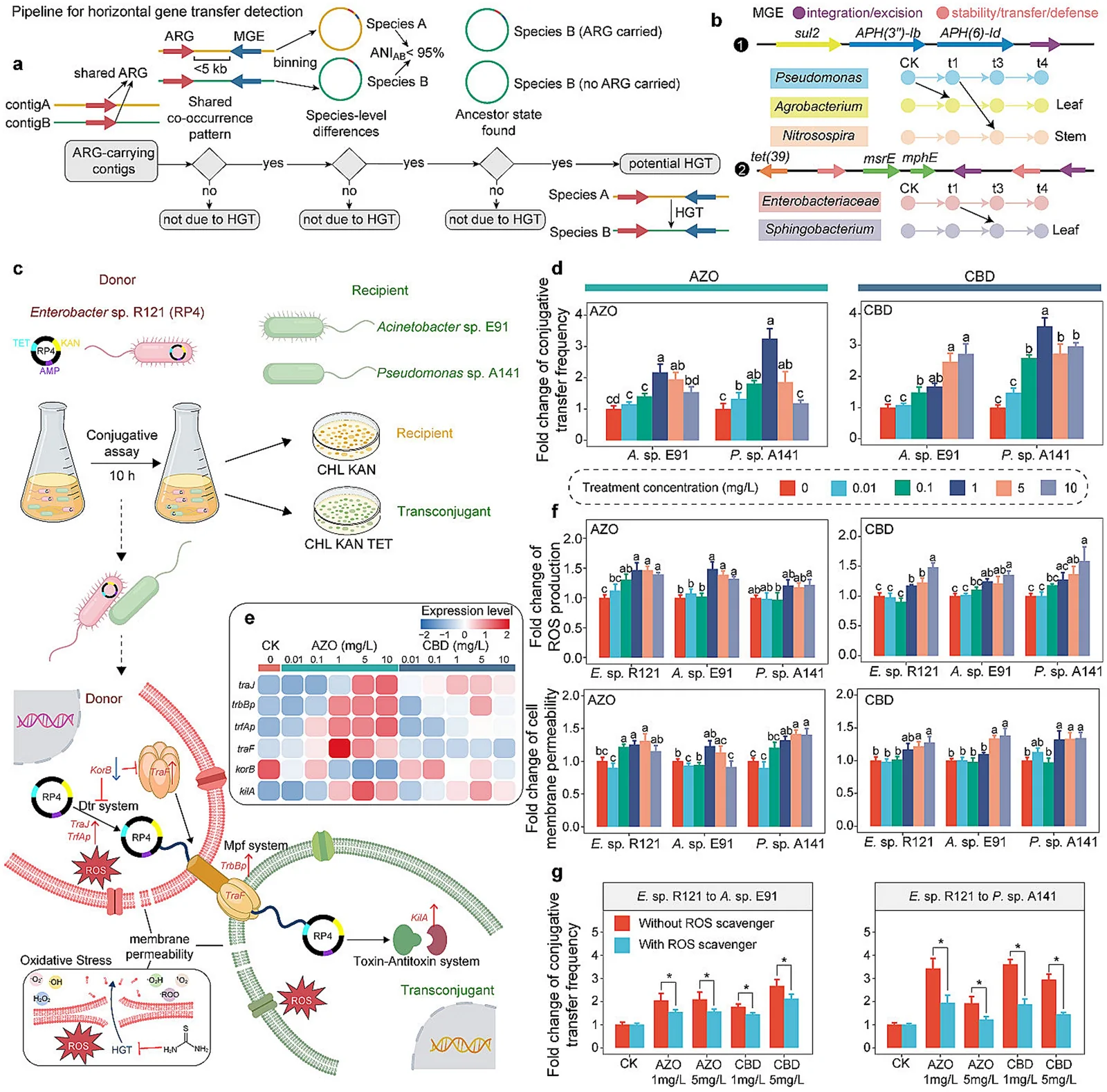
Effect of fungicide stress on the horizontal transfer of ARGs. (**a**) Pipeline for HGT detection of ARG. (**b**) HGT events in the aboveground tissues. t1, t3, and t4 represent samples collected after the first, third, and fourth fungicide applications, respectively, and arrows indicate HGT events. (**c**) Conjugation assay based on indigenous strains isolated from the tomato cultivation system. Specifically, *Enterobacter* sp. R121, carrying plasmid RP4, served as the donor, and *Acinetobacter* sp. E91 and *Pseudomonas* sp. A141 were used as recipients. (**d**) Fold change of conjugative transfer frequency under fungicide stress. (**e**) Effect of fungicides on the expressions of key genes in plasmids. (**f**) Fold change of reactive oxygen species (ROS) production and membrane permeability under fungicide stress. (**g**) Effect of ROS scavenger (thiourea) on conjugative transfer frequency under fungicide stress (significance: ^*^ for *P* < .05). CK: control; AZO: azoxystrobin; CBD: carbendazim.

HGT is a central ecological process driving the resistome assembly. Although several mobile/conjugative plasmids were reconstructed using metaplasmidSPAdes, direct evidence of HGT events remained limited (Supplementary Fig. 12). To address this, we developed a stringent workflow based on metagenome binning (Fig. 6a). Two multidrug-resistant cassettes were identified in the aboveground tissues: *sul2*-*APH(3″)*-*Ib-APH(6)-Id* and *tet(39)*-*msrE*-*mphE*, which were horizontally transferred from *Pseudomonas* and *Enterobacteriaceae* to phylogenetically distinct genera (Fig. 6b).

To evaluate the potential for fungicide-enhanced emergence of multidrug-resistant bacteria, we established a conjugation system using indigenous strains isolated from the tomato cultivation system (Fig. 6c). The plasmid conjugation frequency gradually increased with rising concentrations of fungicide exposure, peaking within the 1–10 mg/L range (Fig. 6d). At these concentrations, fungicide exposure induced the upregulation of key genes associated with the mating pair formation (Mpf) system (*traF* and *trbBp*), DNA transfer and replication (Dtr) system (*traJ* and *trfAp*), and vertical inheritance (*kilA*). Simultaneously, the global regulator *korB*, which negatively regulates conjugative transfer, was significantly downregulated (Fig. 6e). ROS production and increased cell membrane permeability were identified as major facilitators of HGT under fungicide stress, particularly at high concentrations, and ROS scavenging experiments further confirmed that oxidative stress played a crucial role in promoting plasmid-mediated HGT (Fig. 6f, g). These results suggest that fungicides may exacerbate HGT of ARG by modulating bacterial physiological phenotypes and gene transcription profiles, thereby accelerating the emergence of multidrug-resistant bacteria within the plant microbiome.

### Fungicide-induced metabolomic reprogramming in roots mediates resistome assembly

**Figure 7:**
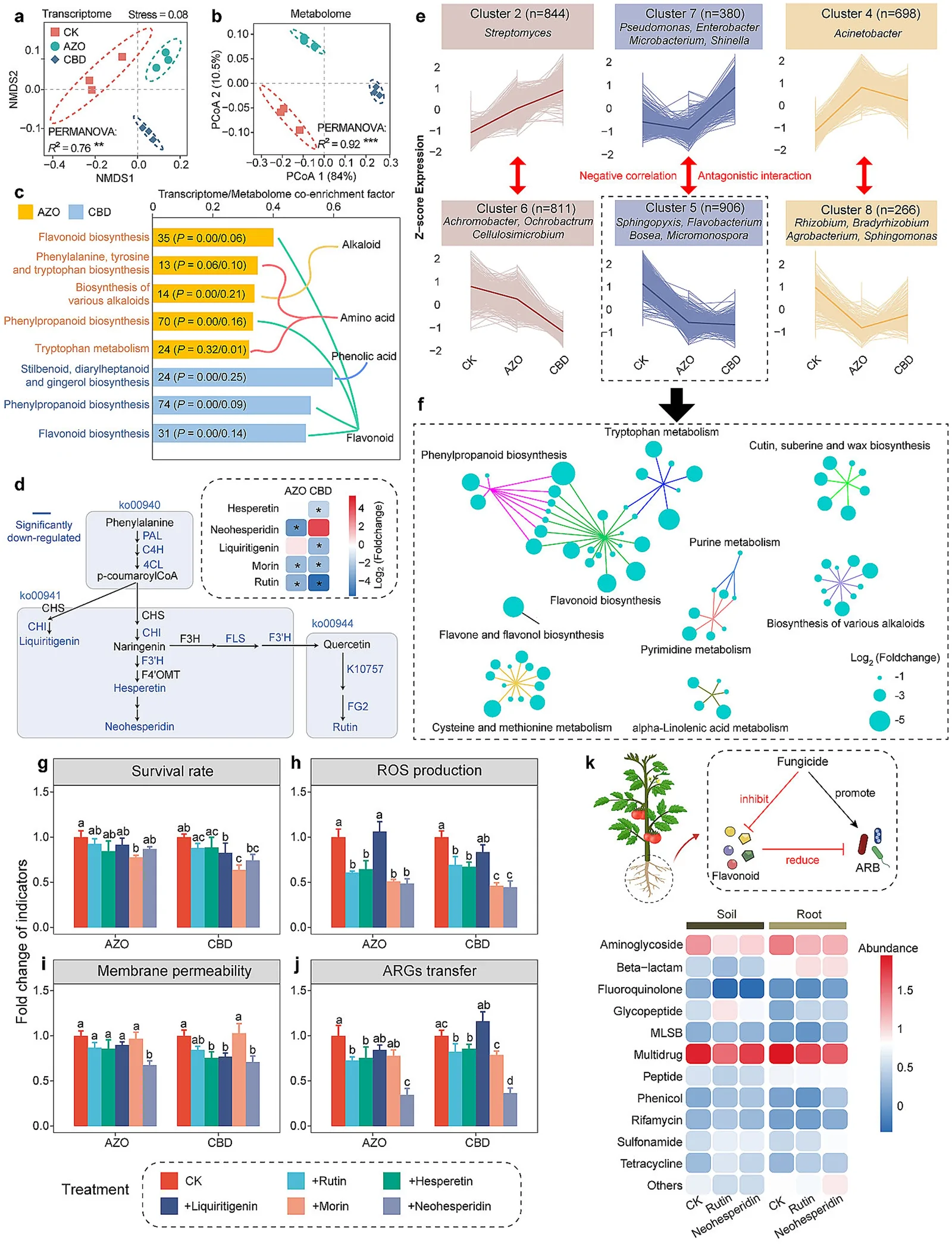
Fungicide-induced transcriptomic and metabolomic reprogramming in roots mediate resistome assembly through multiple ecological processes. **(a**) Non-metric multidimensional scaling (NMDS) of transcriptome under fungicide stress (PERMANOVA: *R*^2^ = 0.76, *P* < .01). (**b**) Principal Co-ordinates Analysis (PCoA) of metabolome under fungicide stress (PERMANOVA: *R*^2^ = 0.92, *P* < .001). (**c**) Transcriptome and metabolome co-enrichment analysis of KEGG pathways under fungicide stress. (**d**) Effect of fungicide on key genes and metabolites in flavonoid biosynthesis-related KEGG pathway of phenylpropanoid biosynthesis (ko00940), flavonoid biosynthesis (ko00941), and flavone and flavonol biosynthesis (ko00944) (significance: ^*^ for *P* < .05). (**e**) K-mean clustering to explore the regulatory network linking genes, metabolites, and microbes (especially antibiotic-resistant bacteria). (**f**) Enriched KEGG pathway of key genes and metabolites in Cluster 5. (g–**j**) Effect of rutin, hesperetin, neohesperidin, morin, and liquiritigenin on survival rate (**g**), reactive oxygen species (ROS) production (**h**), membrane permeability (**i**), and ARG horizontal transfer frequency (**j**) of native ARB in the indigenous ARB-based conjugative transfer model under fungicide stress. (**k**) Effect of rutin and neohesperidin supplementation on the abundance of dominant ARG types in root and rhizosphere soil under fungicide stress. CK: control; AZO: azoxystrobin; CBD: carbendazim.

Fungicide stress induced transcriptomic and metabolomic reprogramming in tomato roots (Fig. 7a,b and Supplementary Figs 13–15). KEGG enrichment analysis indicated that both differentially expressed genes (DEGs) and DAMs were significantly enriched in phenylpropanoid and flavonoid biosynthesis pathways (Fig. 7c). Focusing on the flavonoid biosynthesis pathway, 14 enzyme-coding genes (eg, *CHI*, *FLS*, and *F3’H*) corresponding to nine biosynthesis steps were significantly downregulated (Fig. 7d and Supplementary Fig. 16). Consistently, among 51 detected flavonoid compounds, 31 (60.8%) were significantly downregulated under fungicide stress (Supplementary Table 6).

To further explore the regulatory network linking genes, metabolites, and microbes, all DEGs, DAMs, and dominant microbes were clustered into eight groups using a k-means clustering algorithm (Supplementary Table 7). Three cooperative and three antagonistic modules involving ARB were identified (Fig. 7e). In particular, genes and metabolites in Cluster 5, enriched in flavonoid, alkaloid, and amino acid pathways, were negatively correlated with *Pseudomonas*, *Enterobacter*, *Microbacterium*, and *Shinella* in Cluster 7 (Fig. 7e, f; Pearson, *r* < −0.6, *P* < .05). These metabolites might recruit beneficial microbes such as *Sphingopyxis*, *Flavobacterium*, *Bosea*, and *Micromonospora*, thereby mediating microbial interaction networks between beneficial microbes and ARB.

Based on their fold-change and interactions with ARB under fungicide stress, five flavonoids of rutin, hesperetin, neohesperidin, morin, and liquiritigenin were selected for mechanistic validation using indigenous antibiotic-resistant strains isolated from the tomato cultivation system (Fig. 7d). Inhibition assays showed that morin significantly suppressed the growth of native antibiotic-resistant strains, whereas the other compounds had minimal inhibitory effects (Fig. 7g). Both rutin and neohesperidin demonstrated ROS-scavenging activity and were able to mitigate fungicide-induced increases in membrane permeability, thereby attenuating the promotive effect of fungicide stress on plasmid-mediated HGT of ARG (Fig. 7h-j). To assess the *in situ* function of differentially accumulated flavonoids in resistome assembly, supplementation experiments with rutin and neohesperidin were conducted in fungicide-treated tomato plants. The results demonstrated that rutin and neohesperidin significantly reduced total ARG abundance by 21%–41% in rhizosphere soil and 17%–24% in root, with the greatest decreases (up to 58%) observed for multidrug and aminoglycoside resistance genes (Fig. 7k and Supplementary Fig. 17).

## Discussion

Fungicide-induced promotion of ARG dissemination was substantially stronger in the plant microbiome than in the soil microbiome, likely owing to different fungicide deposition levels and community heterogeneity. Soil microbial communities generally possess higher diversity and stability, and their greater functional redundancy confers enhanced buffering capacity against environmental stress [50]. In contrast, the plant microbiome often exhibits reduced h due to limited nutrient availability, fluctuating environmental conditions, and physical barriers, a phenomenon previously described as “enrichment bias” [51, 52]. The lower community resistance, unique metabolic capacities, and specific resilience mechanisms of the plant microbiome may therefore lead to distinct responses to fungicide stress [53]. Of particular concern, antibiotic-resistant ESKAPE pathogens persisting at low abundances in soil may disseminate via aerosols or migrate through plant vascular systems, ultimately colonizing aboveground tissues [54]. These isolates exhibited widespread resistance to clinically relevant antibiotics, including ampicillin (56.2%), erythromycin (80.9%), and tetracycline (34.8%), consistent with their ARG profiles. Thus, fungicide-induced enrichment of antibiotic-resistant ESKAPE pathogens in the plant microbiome poses a potential risk to public health through the food chain.

Understanding the ecological processes underlying resistome assembly under fungicide stress is essential for identifying the primary pathways of antibiotic resistance dissemination. In the aboveground tissues, the resistome was predominantly shaped by HoS and HD. Strong selection pressure often favors specialized microbes capable of performing specific metabolic functions [55]. HoS-driven ARB typically possessed multifunctional metabolic capacities involving unique carbon, nitrogen, sulfur, and energy metabolism pathways, along with the ability to synthesize antimicrobial compounds. These traits enhance their competitiveness for resources within the plant microbiome, where nutrient scarcity, community simplification, and increased niche overlap may intensify microbial antagonistic interactions under fungicide stress [56]. In contrast, HD-driven ARB relied on two-component systems that respond to multiple environmental signals, representing an adaptive strategy to the extreme and heterogeneous conditions in the phyllosphere [57]. This strategy was consistent with results of the fluorescently labeled ARB migration model, in which HD-driven strains such as *E. ludwigii* and *P. aeruginosa* demonstrated significantly greater upward migration capacity compared to others. In summary, fungicide exposure promoted both selection and dispersal of ARB with distinct metabolic traits, thereby amplifying their ecological risk within the plant microbiome.

Our study also highlights that gene mutation and HGT are often overlooked yet critical ecological processes. Fungicide-induced intensification of positive selection may drive microbial evolution toward acquiring specialized metabolic capabilities, including antibiotic resistance [58]. Enhanced antibiotic resistance can confer adaptive advantages in coping with environmental pollutants and microbial competition through efflux pump mechanisms [59]. Moreover, the aboveground tissues appeared to be hotspots for HGT, where the abundance of MGEs reached 8.05–15.54-fold higher than that in soil. Fungicide stress frequently triggered elevated ROS production and increased cell membrane permeability, both of which are known to facilitate HGT of ARG [6]. Although gene mutation and HGT are inherently stochastic, strong environmental selection and microbial antagonistic interactions can promote the retention of beneficial mutations and HGT, thus transforming stochastic processes into deterministic ones [60].

The rhizosphere constitutes a distinct microenvironment shaped by root exudates. Fungicide exposure can alter microbial communities by disrupting root secretion, thereby modulating plant-microbiome interactions [61]. Based on our flavonoid supplementation experiments, we propose that fungicides induce transcriptomic reprogramming of flavonoid biosynthesis in roots, leading to a significant reduction in flavonoid accumulation. This reduction in flavonoid secretion disrupts redox homeostasis in the rhizosphere, exposing indigenous microbes to intensified oxidative stress. Consequently, elevated intracellular ROS production and increased cell membrane permeability trigger bacterial phenotypic changes, which promote HGT of ARG (Supplementary Fig. 18). Therefore, flavonoids may serve as potential inhibitors of plasmid conjugation, offering a promising strategy for controlling the spread of antibiotic resistance [2].

In conclusion, fungicide-driven selection, dispersal, and HGT facilitated ARG dissemination through multiple interconnected pathways, including root exudate–microbiome interactions, endophytic translocation along the root–stem–leaf continuum, and exchange across the phyllosphere interface. Our work established a comprehensive framework to elucidate the ecological assembly mechanisms of the tomato resistome, underscoring the necessity of regulating fungicide application to mitigate the ecological risks associated with the plant resistome.

## Supplementary Material

Supplementary_material_wrag182

## Data Availability

The sequence data have been uploaded to Sequence Read Archive of NCBI (PRJNA1310417, PRJNA1309026, PRJNA1311277, and PRJNA1311030).
