# Supplementary material for: Bridging ecological processes to elevated antibiotic resistance risk in tomato microbiome under fungicide stress
Source: ISME J. 2026 Jul 12;20(1):wrag182. doi: 10.1093/ismejo/wrag182 (PMC13499529; doi:10.1093/ismejo/wrag182)
Supplement: Supplementary file 1 [file supplementary_material_wrag182.zip › Supplementary_Information_wrag182.docx]

**Supplementary Information**

**Bridging ecological processes to elevated antibiotic resistance risk in tomato microbiome under fungicide stress**

**Contents**

**Supplementary Results**

**Supplementary Result 1.** Succession of the plant microbiome and resistome under fungicide stress in laboratory pot experiments.

**Supplementary Result 2.** Dissipation and translocation characteristics of fungicides in the laboratory/field tomato cultivation system.

**Supplementary Methods**

**Supplementary Method 1.** Chemicals and reagents.

**Supplementary Method 2.** Soil treatment.

**Supplementary Method 3.** Laboratory pot experiment.

**Supplementary Method 4.** Greenhouse experiment.

**Supplementary Method 5.** Fungicide residue extraction.

**Supplementary Method 6.** UPLC-MS/MS conditions for fungicide residue analysis.

**Supplementary Method 7.** Matrix effects, linearity, and limit of quantification (LOQ).

**Supplementary Method 8.** Fortified recovery experiments.

**Supplementary Method 9.** Fungicide residue analysis.

**Supplementary Method 10.** DNA extraction from plant samples and metagenomic sequencing.

**Supplementary Method 11.** Quantification of ARGs by qPCR.

**Supplementary Method 12.** Microbiome analysis.

**Supplementary Method 13.** Isolation of antibiotic-resistant bacteria.

**Supplementary Method 14.** Determination of antibiotic resistance levels.

**Supplementary Method 15.** Bacterial strains and plasmids for construction of fluorescently labeled antibiotic-resistant bacteria (ARB).

**Supplementary Method 16.** Preparation of electrocompetent cells and transformation.

**Supplementary Method 17.** Fluorescent labeling of antibiotic-resistant bacteria.

**Supplementary Method 18.** Verification of fluorescently labeled strains.

**Supplementary Method 19.** Effect of fluorescent labeling on bacterial growth.

**Supplementary Method 20.** Fluorescently labeled antibiotic-resistant bacteria (ARB) migration assay.

**Supplementary Method 21.** Confocal Laser Scanning Microscopy (CLSM) observation.

**Supplementary Method 22.** Quantification of antibiotic-resistant bacteria migration.

**Supplementary Method 23.** Plasmid curing and antibiotic susceptibility testing.

**Supplementary Method 24.** Preparation of plasmid donor strains.

**Supplementary Method 25.** Composition of donor and recipient strains in the conjugative transfer system.

**Supplementary Method 26.** Preparation of donor and recipient bacterial suspensions.

**Supplementary Method 27.** Plasmid conjugative transfer assay.

**Supplementary Method 28.** Quantification of transconjugants and calculation of conjugation transfer frequency.

**Supplementary Method 29.** Quality control and verification of plasmid genetic stability.

**Supplementary Method 30.** Detection of intracellular Reactive Oxygen Species (ROS) in donor and recipient strains.

**Supplementary Method 31.** Assessment of cell membrane permeability.

**Supplementary Method 32.** Gene expression analysis (RT-qPCR).

**Supplementary Method 33.** Tomato cultivation experiment for analysis of root exudate-rhizosphere microbiome interaction.

**Supplementary Method 34.** RNA extraction.

**Supplementary Method 35.** RNA sequencing and transcriptome analysis.

**Supplementary Method 36.** Extraction of tomato root exudates and widely targeted metabolomics analysis.

**Supplementary Figures**

**Supplementary Fig. 1.** Non-metric multidimensional scaling (NMDS) of ARG profiles among different plant tissues and soils based on Bray-Curtis dissimilarity.

**Supplementary Fig. 2.** Effects of fungicide on the absolute abundance of dominant ARGs in the greenhouse tomato cultivation system by quantitative PCR.

**Supplementary Fig. 3.** Effects of fungicide on the abundance of dominant mobile genetic element (MGE) type in tomato tissues and soil.

**Supplementary Fig. 4.** Effects of fungicide on the abundance of dominant virulence factor gene (VFG) type in tomato tissues and soil.

**Supplementary Fig. 5.** The typical co-occurrence pattern of highest-priority ARGs, MGEs, and VFGs in the assembled contigs under fungicide exposure.

**Supplementary Fig. 6.** Co-succession of the microbiome and resistome in plant tissues and soil under fungicide stress.

**Supplementary Fig. 7.** Relative importance of ecological processes to the assembly of antibiotic-resistant bacteria (ARB) and other taxa under fungicide stress in different plant tissues and soil.

**Supplementary Fig. 8.** Effect of fungicide on microbial metabolic traits.

**Supplementary Fig. 9.** Evolutionary tree of metagenome-assembled genomes carrying ARG, MGE, and VFG and their dispersal rate under fungicide stress.

**Supplementary Fig. 10.** Classification and resistance levels of culturable antibiotic-resistant bacteria in tomato cultivation system after application of fungicide.

**Supplementary Fig. 11.** Colonization of representative resistant strain *Pseudomonas* sp. A141-*gfp* in soil and tomato root by confocal laser scanning microscopy (CLSM).

**Supplementary Fig. 12.** Typical mobilizable/conjugative plasmids which were assembled by metaSPAdes and metaplasmidSPAdes.

**Supplementary Fig. 13.** KEGG enrichment analysis of differentially expressed genes in tomato root under the stress of fungicide.

**Supplementary Fig. 14.** Volcano plot of differentially accumulated metabolites in the root exudates of tomato under stress of fungicide.

**Supplementary Fig. 15.** Changes of differentially accumulated metabolites in the root exudates of tomato under stress of fungicide.

**Supplementary Fig. 16.** Differentially accumulated metabolites and differentially expressed genes in flavonoid biosynthesis related pathway under stress of fungicide.

**Supplementary Fig. 17.** Effect of rutin and neohesperidin supplementation on total ARG abundance in tomato root and rhizosphere soil under fungicide stress.

**Supplementary Fig. 18.** A schematic of the mechanism by which carbendazim and azoxystrobin influence the antibiotic resistome in tomato cultivation system through the induction of metabolic reprogramming of flavonoids in tomato root.

**Supplementary Fig. 19.** Effect of fungicide on the abundance of dominant (Top-10) bacterial phyla in tomato tissues and soil.

**Supplementary Fig. 20.** Effect of fungicide on the abundance of dominant (Top-50) bacterial genera in tomato tissues and soil.

**Supplementary Fig. 21.** Effect of fungicide on the total abundance of ARG as well as the abundance of dominant ARG type in tomato tissues and soil.

**Supplementary Fig. 22.** Effect of fungicide on the abundance of dominant (Top-30) ARG subtype in tomato tissues and soil.

**Supplementary Fig. 23.** The types of resistance mechanisms of ARGs in various parts of tomato plant and soil under fungicide stress.

**Supplementary Fig. 24.** Number of shared ARG subtypes in various parts of tomato plant and soil under fungicide stress.

**Supplementary Fig. 25.** Effect of fungicide on the abundance of dominant MGE in various parts of tomato plant and soil.

**Supplementary Fig. 26.** Correlation between ARGs and MGEs abundances in various parts of tomato plant and soil.

**Supplementary Fig. 27.** The typical co-occurrence pattern of ARGs and MGEs in the assembled contigs in laboratory tomato cultivation system under fungicide exposure.

**Supplementary Fig. 28.** Dissipation dynamics of carbendazim and azoxystrobin in the laboratory tomato cultivated soil.

**Supplementary Fig. 29.** Residues of carbendazim and azoxystrobin in root, stem, leaf, and fruit of laboratory-cultivated tomato.

**Supplementary Fig. 30.** Deposition amounts of carbendazim and azoxystrobin in tomato cultivation system.

**Supplementary Fig. 31.** Dissipation dynamics of carbendazim and azoxystrobin in the greenhouse tomato cultivated soil.

**Supplementary Fig. 32.** Dissipation dynamics of carbendazim and azoxystrobin in the greenhouse tomato root.

**Supplementary Fig. 33.** Dissipation dynamics of carbendazim and azoxystrobin in the greenhouse tomato stem.

**Supplementary Fig. 34.** Dissipation dynamics of carbendazim and azoxystrobin in the greenhouse tomato leaf.

**Supplementary Fig. 35.** Dissipation dynamics of carbendazim and azoxystrobin in the greenhouse tomato fruit.

**Supplementary Fig. 36.** Fluorescence verification of *gfp*-labeled antibiotic resistant bacteria (Scale bar was 20 μm).

**Supplementary Fig. 37.** Overlay of total ion chromatograms (TICs) of QC samples (negative (N) ionization mode).

**Supplementary Fig. 38.** Overlay of total ion chromatograms (TICs) of QC samples (positive (P) ionization mode).

**Supplementary Fig. 39.** Distribution of coefficient of variation (CV) among groups.

**Total:**

**Number of results: 2**

**Number of methods: 36**

**Number of figures: 39**

**Supplementary Result 1. Succession of the plant microbiome and resistome under fungicide stress in laboratory pot experiments**

The dominant bacterial phyla in tomato tissues were *Proteobacteria*, *Actinobacteria*, *Bacteroidetes*, and *Firmicutes*, whereas *Actinobacteria*, *Proteobacteria*, *Bacteroidetes*, and *Chloroflexi* predominated in soil (Supplementary Fig. 19). At the genus level, *Sphingomonas*, *Pseudomonas*, *Microbacterium*, *Nocardioides*, and *Rhodococcus* were the most abundant taxa in tomato tissues (Supplementary Fig. 20). Fungicide exposure significantly reshaped the microbial community composition across different plant tissues and soil, with observable spatial heterogeneity (ANOSIM, *P* < 0.05).

The abundance of antibiotic resistance genes (ARGs) in plant tissues and soil increased to 1.04–1.39-fold and 1.02–1.83-fold relative to the control under azoxystrobin and carbendazim stress, respectively, with particularly notable increases observed in fruit and leaf (Supplementary Fig. 21). Fungicide significantly elevated the abundance of multidrug, peptide, aminoglycoside, tetracycline, glycopeptide, and β-lactam resistance genes, and several ARG types showed more than tenfold increases (Supplementary Fig. 21; Wilcoxon signed-rank test, *P* < 0.05). Key ARG subtypes such as *mdtB*, *vanRO*, *vanSO*, *MexF*, *tetA(48)*, *tetB(48)*, and *AAC(6’)-Ib7* were significantly enriched under fungicide stress (Supplementary Fig. 22; Wilcoxon signed-rank test, *P* < 0.05). These genes were primarily associated with resistance mechanisms including antibiotic efflux, target alteration, and inactivation (Supplementary Fig. 23). Additionally, fungicide exposure increased the number of shared ARGs among the root, stem, leaf, fruit, and soil, suggesting potential upward transmission of ARGs driven by fungicide application (Supplementary Fig. 24).

The impact of fungicides on mobile genetic elements (MGEs) in the tomato cultivating system was further investigated. Both azoxystrobin and carbendazim significantly increased the total abundance of MGEs, especially plasmids, in roots and soil (Supplementary Fig. 25; Wilcoxon signed-rank test, *P* < 0.05). Specifically, plasmid abundance reached 1.7- and 1.6-fold (carbendazim), and 1.5- and 2.2-fold (azoxystrobin) that of the control. A strong positive correlation was observed between the abundances of ARGs and MGEs, particularly plasmids, in root, leaf, and soil (Supplementary Fig. 26; adjusted *R*^2^ > 0.85, *P* < 0.05). Several co-localization patterns of representative ARGs and MGEs were identified within contigs, including *tet(A)*, *sul1*, *tolC*, *acrD*, *mdtB*, *bacA*, and *APH(3’)-Ib*. Multiple ARGs (*APH(3’)-Ib*, *acrD*, and *tet(A)*) were co-located with conjugative plasmid-associated MGEs such as *trbJ*, *trbP*, *trfA*, *traA*, and *traC* (e.g., contig NODE_85_length_22730_cov_6.462359; Supplementary Fig. 27). Sequence alignment revealed >99% identity with known broad-host-range conjugative resistance plasmids, including IncP-α plasmids RK2, RP4, and pME8, highlighting the potential role of plasmid-mediated conjugative transfer in the dissemination of ARGs.

**Supplementary Result 2. Dissipation and translocation characteristics of fungicides in laboratory/field tomato cultivation system**

In laboratory cultivation experiments, the dissipation dynamics of carbendazim and azoxystrobin in soil followed first-order kinetic models, exhibiting a rapid dissipation phase initially, followed by a slower phase (Supplementary Fig. 28). The half-lives of carbendazim and azoxystrobin in soil were 12.63 d and 26.76 d, respectively. The distribution of carbendazim in tomato plants followed the order of leaf ≈ stem > root, suggesting an upward translocation and accumulation in the aerial parts (Supplementary Fig. 29). In contrast, the distribution of azoxystrobin followed the order of root > stem ≈ leaf, indicating limited upward mobility and predominant accumulation in the roots (Supplementary Fig. 29).

In field experiments, the highest fungicide deposition occurred in the leaves, which was primarily due to foliar spraying as the application method. Overall, the residue levels in the greenhouse tomato system followed the pattern of leaf/stem > fruit > soil > root for carbendazim, and leaf/stem > root/soil > fruit for azoxystrobin (Supplementary Fig. 30). Increasing application doses and frequencies led to higher residual accumulation of both fungicides in roots, stems, leaves, fruits, and soil (Supplementary Figs. 31-35). The dissipation of carbendazim and azoxystrobin across all plant tissues and soil also conformed to first-order kinetic models, displaying a trend of rapid initial dissipation followed by slower stage (Supplementary Figs. 31-35). The estimated half-lives ranged from 1.99 to 7.75 d for carbendazim and from 2.25 to 17.39 d for azoxystrobin (Supplementary Table 8). The dissipation half-life of carbendazim within plant tissues and soil followed the order: fruit > stem > root > soil > leaf, whereas for azoxystrobin the order was: root > stem > fruit > soil > leaf (Supplementary Table 8).

**Supplementary Method 1. Chemicals and reagents**

Carbendazim (CBD) analytical standard (97.8%) and azoxystrobin (AZO) analytical standard (98.8%) were purchased from Northern Weiye Metrology Group Co., Ltd. (China). Commercial formulations, including 50% carbendazim wettable powder and 250 g L^-1^ azoxystrobin suspension concentrate, were obtained from Jiangsu Lanfeng Bio-Chemical Co., Ltd. (China) and Syngenta Nantong Crop Protection Co., Ltd. (China), respectively. Flavonoid standards (>98% purity) were purchased from MedChemExpress (USA). HPLC-grade methanol and acetonitrile were purchased from Thermo Fisher Scientific (USA). Primary secondary amine (PSA) and graphitized carbon black (GCB) were obtained from Agela Technologies (China). Analytical-grade sodium chloride and anhydrous magnesium sulfate were purchased from Sinopharm Chemical Reagent Co., Ltd. (China).

Phosphate-buffered saline (PBS), Luria–Bertani (LB) broth, and LB agar were purchased from Sangon Biotech (China). Mueller–Hinton (MH), R2A, and tryptic soy agar (TSA) media were obtained from Solarbio Life Sciences (China). Sterile Petri dishes and disposable L-shaped spreaders were purchased from Kodichen Biotechnology Co., Ltd. (China). Murashige and Skoog (MS) medium was obtained from PhytoTech Labs (USA).

The FastDNA Kit for Soil was purchased from MP Biomedicals (USA). PrimeSTAR Max Premix, TaKaRa MiniBEST Plasmid Purification Kit, DL15000 DNA Marker, and DNA Loading Buffer were obtained from Takara Bio Inc. (Japan). PCR-related reagents, including 2×Easy Flash PCR MasterMix, 2×EasyStar Green Fast Mixture, Gel Extraction & Clean-up Kit, and Plasmid Miniprep Kit, were purchased from Sangon Biotech (China). Additional molecular biology reagents, including 2×Taq PCR Master Mix, 2×EasyTaq PCR SuperMix (−dye), EasyPure PCR Purification Kit, pEASY-T1 Simple Cloning Kit, TransStart Top Green qPCR SuperMix, and GelStain nucleic acid stain, were obtained from TransGen Biotech (China). High-purity low-electroendosmosis agarose, DL2000 DNA Marker, and DL10000 DNA Marker were purchased from Tsingke Biotechnology Co., Ltd. (China). MicroAmp Fast Reaction Tubes, MicroAmp Fast Optical 96-Well Reaction Plates, and MicroAmp Optical Adhesive Films were obtained from Thermo Fisher Scientific (USA).

The bacterial genomic DNA extraction kit was obtained from Tiangen Biotech Co., Ltd. (China). The E.Z.N.A. Plasmid DNA Mini Kit I was purchased from Omega Bio-Tek (USA), whereas the HiPure BAC Mini Kit was obtained from Magen Biotechnology Co., Ltd. (China). Plasmid-Safe ATP-Dependent DNase was purchased from Lucigen (USA), and the Zymo Genomic DNA Clean & Concentrator Kit was obtained from Zymo Research (USA). The TransZol Up Plus RNA Kit and TransScript cDNA Synthesis SuperMix were purchased from TransGen Biotech (China).

**Supplementary Method 2. Soil treatment**

Soil used in the pot experiments was collected from an agricultural site in Qingjiangpu District, Huai'an City, Jiangsu Province, China (33°33′N, 118°58′E). Multiple 20 × 20 cm plots were randomly selected, and topsoil samples (0–15 cm depth) were obtained. The soil was classified as silt loam and had no application history of antibiotics and plant protection products including carbendazim and azoxystrobin in the past five years. After air-drying at room temperature, stones, plant roots, and other debris were manually removed. The soil was then sieved through a 2 mm mesh and adjusted to 65% of its water-holding capacity. Following thorough homogenization, the soil was incubated indoors for one month to restore microbial homeostasis before use in subsequent pot experiments. The physicochemical properties of soil for the laboratory pot experiment were: soil type was silt loam, with a pH of 6.3, organic matter content of 30.5 g/kg, total carbon of 1.2%, total nitrogen of 0.16%, total phosphorus of 0.45 g/kg, available nitrogen of 81.2 mg/kg, available phosphorus of 20.6 mg/kg, and cation exchange capacity of 11.6 cmol/kg.

**Supplementary Method 3. Laboratory pot experiment**

The laboratory pot experiment was designed as a preliminary trial to evaluate the effects of the fungicide on the transmission of ARGs within the soil-tomato system under highly controlled conditions. To avoid confounding factors associated with foliar spraying such as spray drift, uneven leaf deposition, and wash-off, we adopted a method in which the fungicide was evenly mixed into the soil. All the results of the laboratory pot experiment are presented in Supplementary Results 1-2, not in the main text. All content in the main text including ARG risk assessment, ecological model construction, and isolation of antibiotic-resistant bacteria is based solely on the greenhouse experiment. The specific procedures of the laboratory pot experiment were outlined as follows:

(1) Seedling cultivation:

Nutrient substrate was evenly distributed into seedling trays, and two cherry tomato seeds were sown per cell. A thin layer of substrate was lightly spread over the seeds, gently compacted, and thoroughly watered. The trays were then covered with plastic film to maintain optimal temperature and humidity for germination. After emergence, seedlings were thinned to retain one healthy plant per cell. During the germination and seedling stages, moisture was consistently maintained. Once the seedlings reached the “four-leaf and one-center” stage, vigorous and uniform plants were transplanted into individual nutrient pots and placed in an artificial climate chamber (set at 25 ± 1°C, with 70% relative humidity and a 16 h light/8 h dark photoperiod) for continued growth for three weeks before use.

(2) Fungicide treatment:

Initial concentrations of carbendazim and azoxystrobin were set at 5.0 mg/kg, based on recommended application doses, reported field residual levels, and preliminary experimental results. Sterile deionized water was added in equal volumes as a control. All treatments were performed in triplicate. Fungicide solutions were sprayed evenly onto the soil using a sprayer and thoroughly mixed. After complete application, soils were placed in a fume hood to allow solvent evaporation. Soil moisture content was adjusted to 65% of its maximum water-holding capacity, homogenized, and passed through a 2 mm mesh sieve. After equilibration, 800 g of treated soil was transferred into each plastic pot (12 cm inner diameter, 14 cm height).

(3) Establishment of the laboratory tomato planting system:

Uniform, healthy cherry tomato seedlings approximately 15 cm in height were selected and transplanted into pots in the morning filled with differently treated soils. Once the leaves had fully unfolded, the pots were placed in a growth chamber set at 25 ± 1°C, with 70% relative humidity and a 16 h light/8 h dark photoperiod. Plant growth and any occurrence of disease or pests were monitored daily. Soil moisture was maintained by periodic weighing and supplementation with sterile deionized water to prevent dehydration and wilting.

(4) Sampling:

Soil samples were collected at 0, 1, 3, 7, 15, and 30 d. Plant tissues (roots, stems, and leaves) were sampled at 3, 7, 15, and 30 d, and fruits were sampled at 30 d. These samples were used to quantify residual concentrations of carbendazim and azoxystrobin in both soil and different plant compartments. Additionally, on day 30, soil and plant samples (leaves, stems, roots, and fruits) were collected for DNA extraction and metagenomic sequencing. All plant and soil samples were stored at −20°C until analysis.

**Supplementary Method 4. Greenhouse experiment**

A greenhouse field experiment was conducted at an experimental greenhouse in Hangzhou, Zhejiang Province, China (30°16′N, 120°12′E). The selected greenhouse (400 m^2^; 50 m × 8 m) had no application history of antibiotics and plant protection products including carbendazim and azoxystrobin in the past five years. Tomatoes were grown in 15 plots, each measuring 24 m^2^ and containing five tomato rows. Buffer rows were established between plots to prevent cross-contamination. During the fruit-setting and harvesting stages, carbendazim and azoxystrobin were applied four times by foliar spraying at 14 d intervals, as detailed in Supplementary Table 9. Tomato plant tissues (leaf, stem, root, and fruit) and soil were sampled at 2 h, 1, 3, 7, and 14 d after each fungicide application. In each plot, plant samples were randomly collected from five representative tomato plants using a five-point sampling method. Soil samples were similarly collected from the 0–15 cm layer using a soil auger. All samples were sealed in sterile polyethylene bags and immediately transported to the laboratory. Tomato tissues and soil samples were stored at –20°C for subsequent fungicide residue analysis and DNA extraction.

**Supplementary Method 5. Fungicide residue extraction**

Fungicide residues in tomato cultivation soil were extracted using the QuEChERS method. Briefly, 5.0 g (dry weight) of soil was weighed into a 50 mL centrifuge tube, followed by the addition of 4 g anhydrous magnesium sulfate, 2 g sodium chloride, and 20 mL acetonitrile. The mixture was homogenized for 3 min using a high-speed disperser, vortexed for 1 min, shaken for 10 min, and ultrasonically extracted for 30 min. The samples were then centrifuged at 8500 r/min for 5 min. A 1.5 mL aliquot of the supernatant was transferred to a new centrifuge tube containing 50 mg PSA and 150 mg anhydrous magnesium sulfate. After vortexing for 45 s and centrifuging at 8500 r/min for 4 min, 0.8 mL of the supernatant was filtered through a 0.22 μm organic membrane and transferred into an autosampler vial for ultra-performance liquid chromatography coupled with tandem mass spectrometry (UPLC-MS/MS) analysis.

Fungicide residues in tomato roots were also extracted using the QuEChERS method. Chopped root samples (2.0 g for greenhouse tomatoes and 0.5 g for laboratory tomatoes) were weighed into 50 mL centrifuge tubes. Each sample was mixed with 4 g anhydrous magnesium sulfate, 2 g sodium chloride, and 20 mL acetonitrile. The homogenization, vortexing, shaking, ultrasonic extraction, and centrifugation steps were identical to those described above. A 1.5 mL portion of the supernatant was transferred to a new tube containing 50 mg PSA and 150 mg anhydrous magnesium sulfate, vortexed for 45 s, centrifuged at 8500 r/min for 4 min, and 0.8 mL of the resulting supernatant was filtered through a 0.22 μm membrane for UPLC-MS/MS analysis.

Fungicide residues in tomato stems, leaves, and fruits were extracted following the same QuEChERS protocol. Chopped plant tissues (5.0 g for greenhouse samples and 2.0 g for laboratory samples) were weighed into 50 mL centrifuge tubes. Each sample was combined with 4 g anhydrous magnesium sulfate, 2 g sodium chloride, and 20 mL acetonitrile. After homogenization for 3 min, vortexing for 1 min, shaking for 10 min, and ultrasonic extraction for 30 min, the mixture was centrifuged at 8500 r/min for 5 min. A 1.5 mL aliquot of the supernatant was transferred to a new centrifuge tube containing 10 mg GCB (except for fruit samples), 50 mg PSA, and 150 mg anhydrous magnesium sulfate. After vortexing for 45 s and centrifugation at 8500 r/min for 4 min, 0.8 mL of the final supernatant was filtered through a 0.22 μm membrane and transferred into an autosampler vial for UPLC-MS/MS analysis.

**Supplementary Method 6. UPLC-MS/MS conditions for fungicide residue analysis**

Fungicide residues were analyzed using ultra-performance liquid chromatography coupled with tandem mass spectrometry (UPLC-MS/MS) under the following conditions:

(1) UPLC conditions:

Chromatographic separation was performed on a Phenomenex Kinetex C18 column (3.0 mm × 100 mm, 2.6 μm particle size). The mobile phase consisted of 0.1% formic acid in water (solvent A) and acetonitrile (solvent B). The gradient elution program was as follows: 0–2.0 min, 90% A; 2.0–5.5 min, 10% A; 5.5–6.5 min, 90% A. The flow rate was set at 0.3 mL/min, the column temperature was maintained at 30°C, and the injection volume was 1 μL.

(2) MS/MS conditions:

Detection was carried out using electrospray ionization in positive mode (ESI+). The nebulizer pressure, 35 psi; drying gas flow rate, 5 L/min; drying gas temperature, 300°C; sheath gas flow rate, 10 L/min; sheath gas temperature, 350°C; capillary voltage, 3.5 kV. The instrument operated in multiple reaction monitoring (MRM) mode. Detailed mass spectrometric parameters are provided in Supplementary Table 10.

**Supplementary Method 7. Matrix effects, linearity, and limit of quantification (LOQ)**

Standard solutions of carbendazim and azoxystrobin were prepared in acetonitrile at concentrations of 0.01, 0.025, 0.050, 0.1, 0.2, and 0.5 mg/L. Blank tomato samples were processed following the procedure described in Supplementary Method 5. The resulting blank sample extracts were then spiked with standard solutions to obtain matrix-matched calibration standards. Linear regression was performed by plotting the analyte peak areas against the corresponding concentrations of the matrix-matched standard solutions. Matrix effects were evaluated by comparing the slopes of calibration curves prepared in matrix-matched extracts versus pure solvent (acetonitrile).

The results showed that both carbendazim and azoxystrobin exhibited good linearity in matrix-matched and solvent-based standard curves over the concentration range of 0.01–0.5 mg/kg. Matrix effects were quantified by comparing the slopes of the matrix-matched and solvent-based calibration curves. Results indicated that both fungicides exhibited matrix enhancement effects. Therefore, external calibration using matrix-matched standards was applied for quantification. The sensitivity of the method was assessed based on the limit of quantification (LOQ) and detection (LOD), which was determined to be 0.01 mg/kg and 0.003 mg/kg, respectively, for both carbendazim and azoxystrobin in all tested matrices, including tomato roots, stems, leaves, fruits, and cultivation soil.

**Supplementary Method 8. Fortified recovery experiments**

To assess method accuracy, blank samples of tomato cultivation soil, leaves, stems, and fruits (5.0 g each), and roots (2.0 g) were spiked with standard solutions of carbendazim and azoxystrobin at final concentrations of 0.01, 0.1, 0.5, and 50 mg/kg. An additional fortifying level of 150 mg/kg was used for leaf samples. Each concentration was analyzed in five replicates. The extraction and quantification of fungicide residues in the fortified samples were conducted following the procedures described in Supplementary Methods 5,6. Recovery rates were calculated based on the detected concentrations of carbendazim and azoxystrobin in each matrix.

The fortified recoveries of both fungicides ranged from 80% to 100% across all matrices and spiking levels, with relative standard deviations (RSDs) below 10%, meeting the criteria of pesticide residue analysis (Supplementary Tables 11,12). Specifically, the recovery rates of carbendazim were 88.2%–95.8% (root), 85.1%–94.5% (stem), 83.1%–95.2% (leaf), 83.7%–92.6 (fruit), and 84.5%–91.7% (soil), and their RSDs were 5.9%–8.8% (root), 4.8%–6.7% (stem), 4.8%–8.5% (leaf), 4.7%–8.1% (fruit), and 5.9%–9.2% (soil), respectively (Supplementary Table 11). The recovery rates of azoxystrobin were 86.8%–94.1% (root), 83.7%–95.8% (stem), 81.7%–95.5% (leaf), 83.2%–90.9% (fruit), and 84.1%–91.1% (soil), and their RSDs were 3.6%–6.2% (root), 4.1%–6.8% (stem), 5.9%–8.2% (leaf), 4.2%–7.5% (fruit), and 4.8%–6.6% (soil), respectively (Supplementary Table 12).

**Supplementary Method 9. Fungicide residue analysis**

Fungicide residues of carbendazim and azoxystrobin in different tomato plant tissues and cultivation soil were quantified using the external standard method, with matrix-matched calibration curves applied for correction. A first-order kinetic model was used to fit the dissipation dynamics of carbendazim and azoxystrobin residues in the tomato plant and soil. The model is described by the following equation:

$$C_{t}=Ae^{-kt}$$

$$t_{1/2}=ln2/k$$

where C_t_ is the concentration of fungicide residue at time t (days), k is the first-order rate constant, A is a constant, and t_1/2_ is half-life.

**Supplementary Method 10. DNA extraction from plant samples and metagenomic sequencing**

Preprocessing of tomato root, stem, leaf, and fruit samples was performed under a laminar flow hood. Specifically, 10 g of leaf, stem, or fruit tissue (or 2 g of root tissue) was weighed and transferred into a sterile beaker. Samples were manually chopped, followed by the addition of 90 mL of sterile 1× PBS (pH 7.0) supplemented with 0.1% glycerol and 0.15% Tween-80. The mixture was homogenized for 2 minutes at medium–low speed using a tissue disperser. The resulting homogenate was sequentially filtered through 150 μm and 26 μm mesh filters to remove large plant debris. The filtered suspension was transferred into 50 mL centrifuge tubes and centrifuged at 8000 r/min for 5 minutes to collect the pellet. The pellet was then washed and resuspended in PBS, followed by centrifugation at 3200 r/min for 10 minutes to remove the supernatant. This washing step was repeated three times by gently rinsing the pellet surface with PBS using a pipette. All wash solutions were pooled and subjected to a final centrifugation at 3200 r/min for 10 minutes. The resulting pellet was retained for total DNA extraction. Depending on sample type and complexity, Nycodenz density gradient centrifugation was applied to purify the microbial fraction when necessary. Total DNA from different plant tissues was subsequently extracted using the FastDNA Spin Kit for Soil (MP Biomedicals, USA), following the manufacturer’s instructions.

Total DNA from different plant and soil samples was extracted using the FastDNA Spin Kit for Soil (MP Biomedicals), following the manufacturer’s instructions. DNA concentration and purity were measured using a micro-volume spectrophotometer. To assess the extent of host DNA contamination, qPCR was performed to determine the relative abundance of the tomato *Actin2* gene versus the bacterial 16S rRNA gene. Only DNA samples that met the following quality criteria were retained and stored at −80°C for further analysis: a tomato *Actin2*-to-bacterial 16S rRNA gene copy number ratio ≤ 0.01, a DNA concentration > 10 ng μL^-1^, and an OD_260_/OD_280_ ratio between 1.8 and 2.0. Metagenomic sequencing was performed by Novogene Co., Ltd. (Tianjin, China). DNA libraries were constructed using the Nextera XT DNA Library Prep Kit (Illumina), with an average insert size of 350 bp (excluding adaptors), and sequenced on the NovaSeq 6000 System (Illumina) using the paired-end 150 bp (PE150) strategy. Raw sequencing data were processed using fastp software to remove low-quality reads, retaining only those with a Phred quality score >20 and fewer than three ambiguous nucleotides. To eliminate host-derived sequences, reads were mapped to the tomato reference genome (NC_015447.3) using Bowtie2 (--end-to-end and --very-sensitive mode) [1]. Only reads that did not align to the host genome were retained for further metagenomic analysis. Summary statistics of the metagenomic datasets are presented in Supplementary Table 13.

**Supplementary Method 11. Quantification of ARGs by qPCR**

The absolute abundances of six dominant ARGs in the tomato cultivation system were quantified using quantitative polymerase chain reaction (qPCR) [2]. The targeted ARGs included three multidrug resistance genes (*mexF*, *mtrA*, and *mdtB*), two glycopeptide resistance genes (*vanRO* and *vanSO*), and one sulfonamide resistance gene (*sul1*). The specific primer sequences used for qPCR analysis are listed in Supplementary Table 14. Each qPCR was carried out in a final volume of 20 μL, and the reaction mixture composition was detailed in Supplementary Table 15. Amplification was performed under the following cycling conditions: initial denaturation at 95°C for 3 minutes, followed by 40 cycles of denaturation at 95°C for 10 seconds, and annealing/extension at 55–60°C for 30 seconds. All reactions were performed in triplicate for each DNA sample to ensure reproducibility.

**Supplementary Method 12. Microbiome analysis**

Taxonomic annotation of metagenomic reads was performed using Kaiju, generating microbial abundance profiles at multiple taxonomic levels. Microbial α-, β-, and γ-diversity were calculated by the R packages "vegan" [3] and "iNEXT" [4]. Microbial niche breadth was calculated by the R package "spaa" [5], and taxa were then categorized into generalists, opportunists, and specialists by the R package "EcolUtils" [6]. Community resistance was defined as the Bray–Curtis similarity in species abundance between control and disturbed communities [7]. Community resistance of generalists, opportunists, and specialists under fungicide stress was calculated, and variance partitioning analysis (VPA) was performed to quantify their contributions to resistome shifts. Co-successional patterns between the microbiome and resistome were evaluated using Procrustes analysis. Microbial source tracking was conducted using the R package "FEAST" [8]. Source and sink communities were defined based on the assumption that microorganisms can migrate through internal pathways along the soil–root–stem–leaf–fruit continuum.

**Supplementary Method 13. Isolation of antibiotic-resistant bacteria**

Antibiotic-resistant bacteria (ARB) from the greenhouse tomato cultivation system were isolated using antibiotic-selective plate culture.

Isolation from soil: Under sterile conditions, 5.0 g of soil was transferred into a sterile Erlenmeyer flask containing 95 mL of sterile phosphate-buffered saline (PBS, pH 7.0). The mixture was shaken at 150 r/min for 60 min at 25°C, then allowed to settle for 30 min. The supernatant was serially diluted to 10^-4^–10^-5^. A 100 μL aliquot of the diluted suspension was spread evenly onto LB, TSA, R2A, or MH agar plates supplemented with 5.0 mg/L of one of the following antibiotics: ampicillin, chloramphenicol, erythromycin, rifampicin, sulfadiazine, or tetracycline. Plates were incubated at 25–30°C for 24–72 h. Colonies with distinct morphologies were picked for streaking and purification. Isolated strains were then identified and preserved for subsequent analyses.

Isolation of epiphytic ARB from tomato tissues: For epiphytic bacteria, 2.0 g of root or 5.0 g of stem, leaf, or fruit tissue was rinsed with sterile water to remove surface debris. Samples were placed into sterile Erlenmeyer flasks with 30 mL sterile PBS, subjected to ultrasonication for 30 min, and shaken at 150 r/min for 2 h at 25°C. The resulting suspension was further processed in the same manner as for soil.

Isolation of endophytic ARB from tomato tissues: For endophytic bacteria, 2.0 g of root or 5.0 g of stem, leaf, or fruit tissue was surface-sterilized by sequential washing: five times with sterile water, followed by 75% ethanol for 1.5 min, and 2.8% sodium hypochlorite for 5 min, then rinsed with sterile water for 1 min. The sterilized tissues were cut into small pieces using sterile scalpels, homogenized, and transferred to sterile flasks containing 30 mL PBS. The homogenates were shaken at 150 r/min for 2 h at 25°C, followed by dilution and plating as described above.

**Supplementary Method 14. Determination of antibiotic resistance levels**

The antibiotic resistance level of each isolated strain was evaluated by determining the minimum inhibitory concentration (MIC). Briefly, bacterial isolates were cultured in Mueller-Hinton (MH) broth until reaching the logarithmic growth phase. Cultures were then adjusted to a 0.5 McFarland turbidity standard using sterile PBS, followed by a 100-fold dilution in MH broth. A two-fold serial dilution of each antibiotic was prepared in MH broth across wells 1–11 of a 96-well microtiter plate, yielding final concentrations ranging from 1 mg/L to 1024 mg/L. Well 12 served as the solvent control. Each well was inoculated with 10 μL of the diluted bacterial suspension. Plates were incubated at 37°C for 16–24 h. Bacterial growth was assessed by visual inspection and measurement of optical density at 600 nm (OD_600_) using a microplate reader. The MIC was defined as the lowest concentration of antibiotic that completely inhibited visible bacterial growth. Three independent biological replicates were performed for each antibiotic against each bacterial strain in the MIC assays. The MIC results for each isolate were interpreted based on the Clinical & Laboratory Standards Institute (CLSI) guidelines [9].

**Supplementary Method 15. Bacterial strains and plasmids for construction of fluorescently labeled antibiotic-resistant bacteria (ARB)**

Bacterial strains: Representative antibiotic-resistant isolates were selected for subsequent experiments. These included *Acinetobacter* sp. E91, *Klebsiella* sp. S21, *Pseudomonas* sp. A141, *Atlantibacter* sp. A31, *Enterobacter* sp. R121, *Pantoea* sp. A32, and *Achromobacter* sp. T121. Phylogenetic analysis based on 16S rRNA gene sequences indicated that these isolates were closely related to *Acinetobacter oryzae* strain B23, *Klebsiella pneumoniae* subsp. rhinoscleromatis strain R-70, *Pseudomonas aeruginosa* strain DSM 50071, *Atlantibacter hermannii* strain CIP 103176, *Enterobacter ludwigii* strain EN-119, *Pantoea agglomerans* strain JCM1236, and *Achromobacter xylosoxidans* strain NBRC 15126, with sequence similarities exceeding 99%. The seven antibiotic-resistant isolates represented the majority of ARB shared between the soil and plant microbiome and were classified as ARB exhibiting a dispersal-driven streamlined metabolic trait. These isolates were therefore selected to validate the contribution of bacterial dispersal to ARG enrichment within the plant microbiome.

Plasmids: The plasmids used in this study included the miniTn7 transposon vector pUC18T-mini-Tn7T-Zeo (5.4 kb, conferring resistance to ampicillin and bleomycin), and a fluorescently labeled derivative, pUC18T-mini-Tn7T-Zeo-*gfp* (5.4 kb, ampicillin and bleomycin resistance), hosted in *Escherichia coli* DH5α. The helper plasmid pTNS2 (9.6 kb, ampicillin resistance) was maintained in *E. coli* SM10(λ pir). The conjugation helper plasmid pRK2013 (48 kb, kanamycin resistance) was hosted in *E. coli* HB101. Additionally, the suicide plasmid pFLP3 (11 kb, ampicillin and tetracycline resistance), which encodes FLP recombinase, was also employed. All plasmid DNA was extracted using the E.Z.N.A. Plasmid DNA Mini Kit I according to the manufacturer’s instructions.

**Supplementary Method 16. Preparation of electrocompetent cells and transformation**

Preparation of electrocompetent cells: Bacterial strains were cultured to early logarithmic phase and harvested by centrifugation at 4°C, 4000 r/min for 10 min. The supernatant was discarded, and the cell pellet was resuspended in approximately 30 mL of ice-cold double-distilled water. The suspension was thoroughly mixed by pipetting and vortexing to ensure homogeneity. Cells were then collected by centrifugation at 6500 r/min for 4 min at 4°C. The washing step was repeated twice with ice-cold 10% glycerol. After the final wash, the pellet was resuspended in 2 mL of 10% glycerol and aliquoted into 80 μL per tube. Aliquots were flash-frozen in liquid nitrogen and stored at −80°C for subsequent electroporation.

Electroporation: For transformation, 2 μL of plasmid DNA was added to 100 μL of thawed electrocompetent cells and gently mixed. The mixture was transferred to a pre-chilled electroporation cuvette, ensuring it settled between the electrodes. Electroporation was performed at 2.5 kV using a standard electroporator. Immediately following the pulse, 1 mL of antibiotic-free LB broth was added to the cuvette to recover the cells, which were then transferred to 1.5 mL microcentrifuge tubes and incubated at 30°C, 180 r/min for 2–4 h. Transformants were selected by plating 100 μL of the recovery culture onto LB agar plates supplemented with the appropriate antibiotics and incubated at 30°C for 24–48 h to observe colony formation.

**Supplementary Method 17. Fluorescent labeling of antibiotic-resistant bacteria**

Antibiotic-resistant isolates were fluorescently labeled using a Tn7-based site-specific chromosomal integration system [10]. Briefly, the green fluorescent protein (*gfp*) gene, including its native promoter, was amplified by PCR and cloned into the mini-Tn7 delivery vector pUC18T-mini-Tn7T-Zeo, yielding pUC18T-mini-Tn7T-Zeo-*gfp*. After sequence verification, the *gfp* cassette was integrated as a single copy into the chromosomal attTn7 site located 25 bp downstream of the conserved *glmS* gene in the resistant bacterial strains [11]. Two strategies were employed for fluorescent labeling: quad-parental conjugation and electroporation.

For quad-parental conjugation, four strains were used: *E. coli* SM10(λpir) harboring the helper plasmid pTNS2, *E. coli* HB101 carrying the mobilization plasmid pRK2013, *E. coli* DH5α harboring the donor plasmid pUC18T-mini-Tn7T-Zeo-*gfp*, and the recipient antibiotic-resistant bacterial isolate. Each strain was pre-cultured overnight in LB broth containing the appropriate antibiotic. Equal volumes (100 μL) of each culture were mixed with 1 mL of antibiotic-free LB to dilute the antibiotic concentration, then centrifuged at 6000 g for 5 min. The pellets were washed three times with LB and resuspended in 30 μL of LB. The mixed suspension was spotted onto the center of an antibiotic-free LB agar plate overlaid with a 0.22 μm sterile membrane filter and incubated at 37°C for approximately 5 h. After incubation, the filter was transferred to a tube containing 5 mL PBS, vortexed thoroughly to release the cells, and the suspension was centrifuged at 6000 g for 5 min. The pellet was resuspended in 0.2 mL PBS, and 100 μL was plated onto selective LB agar containing appropriate antibiotics.

For electroporation, 50 ng each of the suicide vector pUC18T-mini-Tn7T-Zeo-*gfp* and the helper plasmid pTNS2 were added to 100 μL of electrocompetent antibiotic-resistant cells. After incubation on ice for 30 min, the mixture was transferred into a 2-mm gap electroporation cuvette and pulsed at 2.5 kV. Immediately afterward, 1 mL LB broth was added to the cuvette, mixed gently, and transferred to a microcentrifuge tube for recovery at 37°C with shaking for 2 h. The culture was then plated on LB agar supplemented with appropriate antibiotics.

The chromosomal insertion site of the mini-Tn7 element was verified by colony PCR. To remove the antibiotic resistance marker introduced by the fluorescent labeling, the FLP recombinase-encoding plasmid pFLP3 was subsequently introduced to excise the resistance gene via site-specific recombination.

**Supplementary Method 18. Verification of fluorescently labeled strains**

A series of fluorescently labeled antibiotic-resistant strains were successfully obtained. The genetic stability and fluorescence intensity of these strains were confirmed through successive subculturing and fluorescence analysis using both colony-based screening and confocal laser scanning microscopy (CLSM).

Colony fluorescence screening: Single colonies were streaked onto fresh agar plates and incubated. Fluorescence was examined after several generations using a LUYOR-3415RG dual-wavelength portable fluorescence protein excitation lamp. Green fluorescence emission from bacterial colonies was used as an indicator of successful GFP expression.

CLSM observation: Cell suspensions were prepared as water-mounted slides and allowed to settle prior to observation. Fluorescence was examined using CLSM with excitation at 488 nm and emission at 507 nm.

All fluorescently labeled strains exhibited stable and robust green fluorescence under CLSM, with intensities sufficient for detection and subsequent experimental applications (Supplementary Fig. 36).

**Supplementary Method 19. Effect of fluorescent labeling on bacterial growth**

To evaluate whether fluorescent labeling affected the physiology of antibiotic-resistant strains, growth kinetics of *gfp*-labeled and wild-type (WT) strains were compared. Bacterial cultures in the logarithmic growth phase were inoculated into LB medium at a 1.0% (v/v) inoculation ratio (12 h, OD_600_ = 1.0), and incubated at 30°C with shaking at 200 r/min for 24 h. Optical density at 600 nm (OD_600_) was measured every 2 h to monitor growth.

The resulting growth curves revealed no significant differences between *gfp*-labeled strains and their corresponding WT counterparts, indicating that chromosomal insertion of the fluorescent marker imposed no detectable metabolic burden on the resistant strains.

**Supplementary Method 20. Fluorescently labeled antibiotic-resistant bacteria (ARB) migration assay**

To investigate the migration dynamics of fluorescently labeled ARB from soil to tomato plants under selective pressure, a sterilized soil cultivation system was established [12]. The experimental procedure was as follows:

1) Seed sterilization: Uniform, plump tomato seeds were surface-sterilized by sequential washing five times with sterile water, followed by immersion in 75% ethanol for 5 min with agitation for 3 min. The ethanol was removed, and the seeds were rinsed five times with sterile water, then soaked in 2.8% sodium hypochlorite for 10 min. After filtration, seeds were rinsed ten additional times with sterile water, air-dried on sterile filter paper for 5–10 min, and transferred to Petri dishes using sterile tweezers.
2) Seed germination and planting: Seeds were germinated on MS agar medium supplemented with B5 vitamins at 22°C. After 7 d, germinated seeds were aseptically transferred to sterile tissue culture bottles containing 80 g of autoclaved soil. Plants were cultivated under controlled conditions (26/20°C day/night temperature, 16 h light/8 h dark photoperiod). Inoculation was performed after 15 d of growth.
3) Preparation of bacterial inoculum: GFP-labeled ARB strains were cultured from single colonies in LB medium containing the appropriate antibiotics until reaching the logarithmic phase. The bacterial suspension was adjusted to OD_600_ ≈ 1.0, corresponding to ~10^8^ CFUs/mL.
4) Fungicide treatment: Under sterile conditions, sterilized soil was amended with technical-grade carbendazim and azoxystrobin to a final concentration of 5 mg/kg each. Sterile water was added in parallel as a control. Soils were thoroughly mixed before use.
5) ARB inoculation and plant cultivation: The prepared bacterial suspension was added to fungicide-treated soil at 10.0% (v/w), resulting in a final ARB concentration of ~10^7^ CFUs/g dry soil. Each culture bottle received 80 g of this inoculated soil. Tomato seedlings were transplanted into the soil and maintained under growth conditions (26/20°C, 16 h light/8 h dark, 65% relative humidity) for 14 d. Sterile water was added every 2 days to prevent plant wilting.

After 14 d of inoculation, samples of soil, roots, stems, and leaves were collected to assess ARB colonization and quantify their migration and establishment within plant tissues.

**Supplementary Method 21. Confocal Laser Scanning Microscopy (CLSM) observation**

Soil samples were prepared as suspensions prior to observation. Tomato plant tissues were surface-sterilized appropriately, manually sectioned, and subsequently examined. Fluorescently labeled antibiotic-resistant bacteria were visualized in tomato roots, stems, and leaves using CLSM, with an excitation wavelength of 488 nm and an emission wavelength of 507 nm. The microscopy procedure was as follows: the system was initialized, and an appropriate objective lens was selected. The region of interest was centered in the field of view, followed by automatic optical path alignment. Fluorescent probes and scanning modes were selected, preview images were acquired, parameters were optimized, and final image acquisition was completed.

**Supplementary Method 22. Quantification of antibiotic-resistant bacteria migration**

Tomato plant tissues (roots, stems, and leaves) and rhizosphere soil samples were collected for analysis. Plant tissues were first rinsed with sterile water to remove surface debris and allowed to air dry. Fresh weight was recorded. Under aseptic conditions, plant samples were sequentially surface-sterilized by immersion in 70% ethanol for 1 min, 1% sodium hypochlorite solution for 2 min, and 70% ethanol for 1 min, followed by thorough rinsing with sterile water to eliminate residual disinfectants. The disinfected tissues were then finely chopped, homogenized, filtered, and centrifuged at 6000 r/min for 5 min to collect the precipitate for subsequent analysis.

To quantify the colonization and translocation of antibiotic-resistant bacteria in tomato tissues and soil, absolute quantification was performed using qPCR, targeting the fluorescent marker gene *gfp* to track bacterial abundance in different plant compartments and soil. The workflow included extraction of genomic DNA from the fluorescently labeled resistant strain and preparation of a standard plasmid containing a single copy of the *gfp* gene. The plasmid concentration was measured to calculate the gene copy number per microliter and generate a standard curve. Total DNA was extracted from plant tissues and soil, followed by qPCR analysis to determine *gfp* copy numbers in each sample. These values were then used to estimate colony-forming units (CFUs) and calculate the abundance of antibiotic-resistant bacteria per gram of dry soil or plant tissue. Detailed qPCR procedures are described in Supplementary Method 11.

**Supplementary Method 23. Plasmid curing and antibiotic susceptibility testing**

To simulate HGT events in environments such as the phyllosphere microbiome, which are often characterized by reduced community complexity, enrichment of antibiotic-resistant bacteria, and elevated abundances of mobile genetic elements that may facilitate microbial gene exchange, a simplified conjugation model was established. To establish a conjugative transfer system based on indigenous bacteria from the tomato cultivation system, plasmids potentially carried by antibiotic-resistant strains were eliminated using the sodium dodecyl sulfate (SDS) curing method. Antibiotic resistance levels of the cured strains were re-evaluated following the procedure described in Supplementary Method 13. The MICs of tested antibiotics are summarized in Supplementary Table 16.

All tested strains exhibited susceptibility to tetracycline (TET). *Enterobacter* sp. R121 displayed resistance only to ampicillin (AMP), whereas *Acinetobacter* sp. E91 and *Pseudomonas* sp. A141 showed resistance to multiple antibiotics, including ampicillin (AMP), chloramphenicol (CHL), and kanamycin (KAN), and exhibited partial resistance to streptomycin (STR) as well.

**Supplementary Method 24. Preparation of plasmid donor strains**

Following the protocol described in Supplementary Method 15, the indigenous tetracycline-sensitive strain *Enterobacter* sp. R121 isolated from the tomato cultivation system was prepared as a competent cell. Plasmids RP4 was subsequently introduced into the strain to construct plasmid donor strain *Enterobacter* sp. R121 (RP4).

**Supplementary Method 25. Composition of donor and recipient strains in the conjugative transfer system**

To evaluate the potential for the emergence of multidrug-resistant bacteria, a conjugative transfer system was established using indigenous bacterial strains isolated from the tomato cultivation system. Based on the MIC profiles of four tested strains, *Enterobacter* sp. R121 (RP4) was designated as plasmid donor strain, and *Acinetobacter* sp. E91 and *Pseudomonas* sp. A141 were selected as recipient strains. Tetracycline (TET) was used to selectively identify plasmid donor strains, whereas chloramphenicol (CHL) and kanamycin (KAN) were used to select recipient strains. For transconjugant selection, a combination of TET, CHL, and KAN was applied. Additionally, ampicillin (AMP) was included in the culture medium to effectively prevent contamination during bacterial cultivation.

**Supplementary Method 26. Preparation of donor and recipient bacterial suspensions**

Donor and recipient strains were cultured overnight in LB medium supplemented with the appropriate antibiotics. Following incubation, cultures were transferred to sterile centrifuge tubes and centrifuged at 6500 r/min for 5 minutes to collect the bacterial pellets. The pellets were washed twice with 30 mL of sterile phosphate-buffered saline (PBS), and the final cell suspensions were adjusted to an optical density at 600 nm (OD_600_) of 0.4.

**Supplementary Method 27. Plasmid conjugative transfer assay**

Equal volumes (2 mL each) of donor and recipient bacterial suspensions were mixed in sterile 10 mL glass tubes. Standard solutions of the fungicides carbendazim and azoxystrobin (dissolved in DMSO) were added to achieve final concentrations of 0.01, 0.1, 1, 5, and 10 mg/L. Control treatments included the addition of an equivalent volume of sterile PBS or DMSO alone. Each treatment was performed in triplicate. After thorough mixing, the conjugation mixtures were incubated statically at 30°C for 10 hours of plasmid transfer [13].

**Supplementary Method 28. Quantification of transconjugants and calculation of conjugation transfer frequency**

Transconjugants were selected using LB agar plates supplemented with a combination of antibiotics. After thorough mixing, conjugation mixtures were serially diluted with sterile water to appropriate concentrations. Then, 50 μL of each dilution was spread onto selective agar plates for transconjugants (containing 20 mg/L chloramphenicol, 60 mg/L kanamycin, 100 mg/L ampicillin, and 10 mg/L tetracycline) and for recipient cells (containing 20 mg/L chloramphenicol, 60 mg/L kanamycin, and 100 mg/L ampicillin). All treatments were performed in triplicate. Plates were incubated at 30°C for 48 hours, after which colony-forming units (CFUs) of both transconjugants and recipient cells were enumerated. The number of transconjugants and recipients was calculated by multiplying the CFU counts by the corresponding dilution factors. Conjugation transfer frequency (F) was determined using the following formula:

F = C_T_ / C_R_

where C_T_ represents the number of transconjugants (CFUs/mL), and C_R_ represents the number of recipient cells (CFUs/mL).

**Supplementary Method 29. Quality control and verification of plasmid genetic stability**

To eliminate the influence of spontaneous mutations, 2 mL of prepared donor and recipient cultures were each mixed with 2 mL of PBS and exposed to the same concentrations of carbendazim and azoxystrobin as used in the conjugation experiments. The mixtures were incubated statically at 30°C for 10 hours. Then, 50 μL of each culture was plated on LB agar supplemented with the corresponding antibiotics for selective growth. To assess the genetic stability of conjugative resistance plasmids, single transconjugant colonies were randomly selected and transferred onto LB agar plates containing 20 mg/L chloramphenicol, 60 mg/L kanamycin, 100 mg/L ampicillin, and 10 mg/L tetracycline. The transconjugants were continuously subcultured on these selective plates for at least five generations. Antibiotic susceptibility testing was performed to confirm the stable inheritance of plasmid-borne resistance traits.

The results demonstrated that transconjugants, including *Acinetobacter* sp. E91 (RP4) and *Pseudomonas* sp. A141 (RP4), maintained stable growth on selective LB agar plates after 4–5 successive generations. Antibiotic susceptibility profiles confirmed that the tetracycline resistance plasmid RP4 was successfully transferred and stably maintained in the transconjugant strains (Supplementary Table 17).

**Supplementary Method 30. Detection of intracellular Reactive Oxygen Species (ROS) in donor and recipient strains**

Intracellular ROS levels in donor and recipient bacteria were quantified using flow cytometry. Bacterial suspensions were prepared following the method described in Supplementary Method 26 to a final concentration of approximately 1.0 × 10^6^ CFUs/mL. A fluorescent ROS probe (DCFH-DA) was added to the suspension at a final concentration of 10 μM, followed by incubation at 37°C for 30 minutes in the dark. The mixture was gently agitated every 3–5 minutes to ensure sufficient contact between the probe and bacterial cells. After incubation, cells were collected by centrifugation at 6000 r/min, and the supernatant was discarded. The pellet was washed three times with PBS to remove excess extracellular probe that had not entered the cells. The washed bacterial suspension was then adjusted to a concentration of 1.0 × 10^6^ CFUs/mL. For ROS induction, 200 μL of the prepared suspension was transferred to sterile 2 mL centrifuge tubes, and exposed to carbendazim and azoxystrobin at final concentrations of 0, 0.01, 0.1, 1, 5, and 10 mg/L. Each treatment was performed in triplicate and incubated at 30 ℃ for 2 hours in the dark. A 100 μM hydrogen peroxide (H_2_O_2_) treatment was included as a positive control. Fluorescence intensity was measured using a CytoFLEX flow cytometer (Ex/Em = 488/525 nm). Each measurement was repeated three times. Data analysis was performed using CytExpert 2.4 software. The relative ROS production was calculated as the fold change in fluorescence intensity of fungicide-treated samples compared to their respective negative controls.

**Supplementary Method 31. Assessment of cell membrane permeability**

Bacterial suspensions (1 mL) of donor and recipient strains were transferred into centrifuge tubes and exposed to carbendazim and azoxystrobin at concentrations identical to those used in the conjugation assays. Each treatment was performed in triplicate. After gentle mixing, the cultures were incubated statically at 30°C for 2 hours. The bacterial concentration was then adjusted to 1.0 × 10^6^ CFUs/mL. For membrane permeability assessment, 2 μL of propidium iodide (PI; 50 μM) was added to 0.1 mL of each suspension. The samples were incubated for 25 minutes at 30°C in the dark. PI-positive cells were quantified using a CytoFLEX flow cytometer (Ex/Em = 561/610 nm) to determine the proportion of cells with compromised membrane integrity under fungicide exposure. All measurements were performed in triplicate. Data were analyzed using CytExpert 2.4 software [14].

**Supplementary Method 32. Gene expression analysis (RT-qPCR)**

Total RNA was extracted from the bacterial cultures of the conjugation system using the TransZol Up Plus RNA Kit. RNA purity and concentration were assessed, and only samples meeting quality standards were stored at −80°C. Reverse transcription reactions were prepared according to the conditions listed in Supplementary Table 18. The reaction mixtures were incubated at 42°C for 15 minutes, followed by heat inactivation at 85°C for 5 seconds, then immediately chilled on ice. The resulting cDNA was quantified for purity and concentration, and stored at −20°C until further analysis. To quantify bacterial molecular responses under carbendazim and azoxystrobin stress during conjugation, six representative functional genes were selected for RT-qPCR analysis. These included genes involved in the mating pair formation system (*Mpf*), *traF* and *trbBp*; the DNA transfer and replication system (*Dtr*), *traJ* and *trfAp*; the global regulator *korB*; and the vertical inheritance-associated gene *kilA*. Some primers were designed based on previous studies [13, 15]. All primers were synthesized by Tsingke Biotechnology Co., Ltd. (Hangzhou, China). The RT-qPCR procedures were conducted according to the protocol described in Supplementary Method 11.

**Supplementary Method 33. Tomato cultivation experiment for analysis of root exudate-rhizosphere microbiome interaction**

Tomato seedlings were cultivated following the method described in Supplementary Method 3, and a pot-based planting system was established. Three treatments were applied: a control, a 5 mg/kg carbendazim treatment, and a 5 mg/kg azoxystrobin treatment, each with three biological replicates. During the experiment, tomato plants were maintained in a controlled growth chamber under a 12 h light/12 h dark photoperiod, at 25°C/15°C (day/night) and 70% relative humidity. After 14 days of exposure, tomato samples were collected. Rhizosphere soil was obtained by gently brushing off the bulk soil attached to roots. Subsequently, the roots were moderately rinsed with sterile water to facilitate the collection of root exudates. After rinsing thoroughly with running water, the roots were washed several times with RNase-free water. Root tissues were then excised using a sterile scalpel, blotted dry with absorbent paper, placed into sterile centrifuge tubes, flash-frozen in liquid nitrogen, and stored at −80°C for further analysis.

**Supplementary Method 34. RNA extraction**

Tomato root samples were ground into a fine powder in a pre-chilled mortar containing liquid nitrogen. The resulting powder was transferred to centrifuge tubes for RNA extraction using the FastPure Universal Plant Total RNA Isolation Kit, following the manufacturer’s protocol. RNA quality was assessed by agarose gel electrophoresis. RNA concentration and integrity were further evaluated using a Qubit 4.0 fluorometer and a Qsep400 bioanalyzer.

**Supplementary Method 35. RNA sequencing and transcriptome analysis**

Messenger RNA (mRNA) was enriched using Oligo(dT) magnetic beads, randomly fragmented into short pieces, and reverse-transcribed into cDNA. Following PCR amplification, sequencing libraries were generated. After library quality assessment, high-throughput sequencing was performed on the HiSeq System (Illumina, NovaSeq6000, USA) by Metware Biotechnology Co., Ltd. (Wuhan, China). Each treatment included three biological replicates. Raw sequencing reads were subjected to quality control using fastp to obtain high-quality clean data for downstream analyses. Detailed Transcriptome data statistics are summarized in Supplementary Table 19. Clean reads were aligned to the tomato reference genome (NC_015447.3) using HISAT2 [16]. Gene-level read counts were quantified using featureCounts, and gene expression levels were normalized as fragments per kilobase of transcript per million mapped reads (FPKM). KEGG pathway enrichment analysis was performed using KOBAS [17]. Differential gene expression analysis was conducted using DESeq2. Genes and metabolites were considered differentially expressed or accumulated (DEGs and DAMs) if they met the criteria of |log_2_FoldChange| ≥ 1 and adjusted *P* < 0.05. To elucidate the genetic regulatory networks driving changes in the rhizosphere microbiome, a correlation network among DEGs, DAMs, and microbes was constructed using k-means clustering by the R package "cluster" [18].

**Supplementary Method 36. Extraction of tomato root exudates and widely targeted metabolomics analysis**

Root exudate samples were retrieved from −80°C storage and thawed at room temperature, followed by vortexing for 30 seconds to ensure homogeneity. An appropriate amount of each sample was vacuum freeze-dried. The dried material was reconstituted at a 30× concentration using 70% methanol containing internal standards. Samples were vortexed for 15 minutes, sonicated in an ice-water bath for 10 minutes, and centrifuged at 12,000 r/min for 3 minutes at 4°C. The resulting supernatant was filtered through a 0.22 μm microporous membrane and transferred to autosampler vials for analysis. Widely targeted metabolomic profiling was performed using UPLC–MS/MS. The metabolomics data were acquired using an Ultra Performance Liquid Chromatography (UPLC) system (ExionLC AD, SCIEX, China) coupled with tandem mass spectrometry (MS/MS).

Chromatographic conditions were as follows: Column: Agilent SB-C18 (1.8 μm, 2.1 mm × 100 mm); Mobile phase: Solvent A was ultrapure water containing 0.1% formic acid; Solvent B was acetonitrile containing 0.1% formic acid; Elution gradient: 0.00 min, 5% B; linear increase to 95% B over 9.00 min; maintained at 95% B for 1 min; returned to 5% B from 10.00–11.10 min and equilibrated at 5% B until 14.00 min; Flow rate: 0.35 mL/min; Column temperature: 40°C; Injection volume: 2 μL.

Mass spectrometry conditions were as follows: Ionization: Electrospray ionization (ESI); ESI temperature: 550°C; Ion spray voltage: +5500 V (positive mode) / –4500 V (negative mode); Gas settings: GS1 = 50 psi; GS2 = 60 psi; Curtain gas (CUR) = 25 psi; Collision-induced dissociation (CID) parameter: High; Scanning mode: Multiple reaction monitoring (MRM), with nitrogen as the collision gas set to medium intensity; Declustering potential (DP) and collision energy (CE) were optimized for each MRM transition. A specific set of MRM transitions was monitored during the corresponding elution time for each metabolite.

Metabolite identification and quantification were performed using the self-built MetWare database (MWDB). During analysis, isotopic signals, redundant adducts (e.g., K⁺, Na⁺, NH₄⁺), and fragment ions originating from larger molecules were excluded. Metabolite quantification was based on the MRM mode using a triple quadrupole mass spectrometer [19]. Raw mass spectrometry data were processed using Analyst 1.6.3 software. Total ion chromatogram (TIC) overlays of quality control (QC) samples showed high consistency in retention time and peak intensity, indicating excellent instrument stability (Supplementary Figs. 37,38). The coefficient of variation (CV), defined as the ratio of the standard deviation to the mean, was used to assess data dispersion. The empirical cumulative distribution function (ECDF) was applied to evaluate the proportion of metabolites with CV values below reference thresholds. Over 85% of detected metabolites in QC samples had CV < 0.5, and more than 75% had CV < 0.3, indicating highly stable and reproducible metabolomics data (Supplementary Fig. 39).

**Supplementary Fig. 1.** Non-metric multidimensional scaling (NMDS) of ARG profiles among different plant tissues and soils based on Bray-Curtis dissimilarity (significance: *** for *P* < 0.001).

**Supplementary Fig. 2.** Effects of fungicide on the absolute abundance of dominant ARGs in the greenhouse tomato cultivation system by quantitative PCR. AZO1: recommended of azoxystrobin; AZO2: double of azoxystrobin; CBD1: recommended of carbendazim; CBD2: double of carbendazim.

**Supplementary Fig. 3.** Effects of fungicide on the abundance of dominant mobile genetic element (MGE) type in tomato tissues and soil. Effects of fungicide on the abundance of dominant MGE type of plasmid (**a**) and phage (**b**) in tomato tissues and soil (significance: * for *P* < 0.05, ** for *P* < 0.01). AZO: azoxystrobin; CBD: carbendazim.

**Supplementary Fig. 4.** Effects of fungicide on the abundance of dominant virulence factor gene (VFG) type in tomato tissues and soil. Effects of fungicide on the abundance of adherence (**a**), nutritional/metabolic factor (**b**), motility (**c**), biofilm (**d**), and antimicrobial activity/competitive advantage (**e**) related VFG type in tomato tissues and soil (significance: * for *P* < 0.05, ** for *P* < 0.01). AZO: azoxystrobin; CBD: carbendazim.

**Supplementary Fig. 5.** The typical co-occurrence pattern of highest-priority ARGs, MGEs, and VFGs in the assembled contigs under fungicide exposure.

**Supplementary Fig. 6.** Co-succession of the microbiome and resistome in plant tissues and soil under fungicide stress. **a**, Procrustes analysis between the bacterial community and resistome (significance: *** for *P* < 0.001). **b**, γ-diversity of the microbiome in different plant tissues and soil. **c**, Principal co-ordinates analysis (PCoA) of the microbiome in different plant tissues and soil. **d**, Community resistance of generalists and other taxa (specialists and opportunists) and their contribution to the resistome by variance partitioning analysis (VPA) in plant tissues and soil under fungicide stress (significance: * for *P* < 0.05, *** for *P* < 0.001).

**Supplementary Fig. 7.** Relative importance of ecological processes to the assembly of antibiotic-resistant bacteria (ARB) and other taxa under fungicide stress in different plant tissues and soil.

**Supplementary Fig. 8.** Effect of fungicide on microbial metabolic traits. **a**, Effect of fungicide on r-/K- strategy ratio of the microbiome in plant tissues and soil (significance: * for *P* < 0.05, *** for *P* < 0.001). **b**, Non-metric multidimensional scaling (NMDS) of functional profile under fungicide stress. **c**, Effect of fungicide on microbial nucleotide diversity (significance: *** for *P* < 0.001). **d**, Effect of fungicide on linkage disequilibrium (D’) which characterized homologous recombination. AZO: azoxystrobin; CBD: carbendazim.

**Supplementary Fig. 9.** Evolutionary tree of metagenome-assembled genomes carrying ARG, MGE, and VFG and their dispersal rate under fungicide stress (Pls: Plasmid, IS: Insertion Sequence, Ph: phage, IGE: Integrative Element). AZO: azoxystrobin; CBD: carbendazim; FEAST: fast expectation-maximization for microbial source tracking.

**Supplementary Fig. 10.** Classification and resistance levels of culturable antibiotic-resistant bacteria in tomato cultivation system after application of fungicide. AMP: ampicillin; CHL: chloramphenicol; ERY: erythromycin; RFP: rifampicin; SDZ: sulfadiazine; TET: tetracycline; AZO: azoxystrobin; CBD: carbendazim.

**Supplementary Fig. 11.** Colonization of representative resistant strain *Pseudomonas* sp. A141-*gfp* in soil (a) and tomato root (b) by confocal laser scanning microscopy (CLSM).

**Supplementary Fig. 12.** Typical mobilizable/conjugative plasmids which were assembled by metaSPAdes (**a**) and metaplasmidSPAdes (**b**).

**Supplementary Fig. 13.** KEGG enrichment analysis of differentially expressed genes (DEGs) in tomato root under the stress of fungicide. Transcriptome revealed 2,433 and 2,341 DEGs in AZO and CBD treatments compared to the control, respectively. AZO: azoxystrobin; CBD: carbendazim.

**Supplementary Fig. 14.** Volcano plot of differentially accumulated metabolites (DAMs) in the root exudates of tomato under stress of fungicide. Metabolomic profiling of root exudates across all samples identified a total of 1,086 metabolites. 37 and 7 DAMs were upregulated, whereas 217 and 652 DAMs were downregulated in the AZO and CBD treatments compared to the control, respectively. AZO: azoxystrobin; CBD: carbendazim.

**Supplementary Fig. 15.** Changes of differentially accumulated metabolites in the root exudates of tomato under stress of fungicide. The downregulated metabolites primarily included alkaloids, flavonoids, amino acids, and organic acids. AZO: azoxystrobin; CBD: carbendazim.

**Supplementary Fig. 16.** Differentially accumulated metabolites and differentially expressed genes in flavonoid biosynthesis related pathway under stress of fungicide. AZO: azoxystrobin; CBD: carbendazim.

**Supplementary Fig. 17.** Effect of rutin and neohesperidin supplementation on total ARG abundance in tomato root and rhizosphere soil under fungicide stress.

**Supplementary Fig. 18.** A schematic of the mechanism by which carbendazim and azoxystrobin influence the antibiotic resistome in tomato cultivation system through the induction of metabolic reprogramming of flavonoids in tomato root.

**Supplementary Fig. 19.** Effect of fungicide on the abundance of dominant (Top-10) bacterial phyla in tomato tissues and soil. AZO: azoxystrobin; CBD: carbendazim.

**Supplementary Fig. 20.** Effect of fungicide on the abundance of dominant (Top-50) bacterial genera in tomato tissues and soil. AZO: azoxystrobin; CBD: carbendazim.

**Supplementary Fig. 21.** Effect of fungicide on the total abundance of ARG as well as the abundance of dominant ARG type in tomato tissues and soil. AZO: azoxystrobin; CBD: carbendazim.

**Supplementary Fig. 22.** Effect of fungicide on the abundance of dominant (Top-30) ARG subtype in tomato tissues and soil. AZO: azoxystrobin; CBD: carbendazim.

**Supplementary Fig. 23.** The types of resistance mechanisms of ARGs in various parts of tomato plant and soil under fungicide stress. AZO: azoxystrobin; CBD: carbendazim.

**Supplementary Fig. 24.** Number of shared ARG subtypes in various parts of tomato plant and soil under fungicide stress. AZO: azoxystrobin; CBD: carbendazim.

**Supplementary Fig. 25.** Effect of fungicide on the abundance of dominant MGE in various parts of tomato plant and soil. AZO: azoxystrobin; CBD: carbendazim.

**Supplementary Fig. 26.** Correlation between ARGs and MGEs abundances in various parts of tomato plant and soil. AZO: azoxystrobin; CBD: carbendazim.

**Supplementary Fig. 27.** The typical co-occurrence pattern of ARGs and MGEs in the assembled contigs in laboratory tomato cultivation system under fungicide exposure.

**Supplementary Fig. 28.** Dissipation dynamics of carbendazim and azoxystrobin in the laboratory tomato cultivated soil. AZO: azoxystrobin; CBD: carbendazim.

**Supplementary Fig. 29.** Residues of carbendazim and azoxystrobin in root, stem, leaf, and fruit of laboratory-cultivated tomato. AZO: azoxystrobin; CBD: carbendazim.

**Supplementary Fig. 30.** Deposition amounts of carbendazim and azoxystrobin in tomato cultivation system. AZO: azoxystrobin; CBD: carbendazim.

**Supplementary Fig. 31.** Dissipation dynamics of carbendazim and azoxystrobin in the greenhouse tomato cultivated soil. AZO: azoxystrobin; CBD: carbendazim.

**Supplementary Fig. 32.** Dissipation dynamics of carbendazim and azoxystrobin in the greenhouse tomato root. AZO: azoxystrobin; CBD: carbendazim.

**Supplementary Fig. 33.** Dissipation dynamics of carbendazim and azoxystrobin in the greenhouse tomato stem. AZO: azoxystrobin; CBD: carbendazim.

**Supplementary Fig. 34.** Dissipation dynamics of carbendazim and azoxystrobin in the greenhouse tomato leaf. AZO: azoxystrobin; CBD: carbendazim.

**Supplementary Fig. 35.** Dissipation dynamics of carbendazim and azoxystrobin in the greenhouse tomato fruit. AZO: azoxystrobin; CBD: carbendazim.

**Supplementary Fig. 36.** Fluorescence verification of *gfp*-labeled antibiotic resistant bacteria (Scale bar was 20 μm).

**Supplementary Fig. 37.** Overlay of total ion chromatograms (TICs) of QC samples (negative (N) ionization mode).

**Supplementary Fig. 38.** Overlay of total ion chromatograms (TICs) of QC samples (positive (P) ionization mode).

**Supplementary Fig. 39.** Distribution of coefficient of variation (CV) among groups.

**References**

(1) Song JJ, Zheng CL, Qiu MT. et al. Mechanisms underlying the overlooked chiral fungicide-driven enantioselective proliferation of antibiotic resistance in earthworm intestinal microbiome. *Environ Sci Technol* 2024;**58**:2931–43. https://doi.org/10.1021/acs.est.3c07761
(2) Song JJ, Li TX, Zheng ZR. et al. Carbendazim shapes microbiome and enhances resistome in the earthworm gut. *Microbiome* 2022;**10**:63. https://doi.org/10.1186/s40168-022-01261-8
(3) Dixon P. VEGAN, a package of R functions for community ecology. *J Veg Sci* 2003;**14**:927–30. https://doi.org/10.1111/j.1654-1103.2003.tb02228.x
(4) Hsieh TC, Ma KH, Chao A. iNEXT: an R package for rarefaction and extrapolation of species diversity (Hill numbers). *Methods Ecol Evol* 2016;**7**:1451–6. https://doi.org/10.1111/2041-210X.12613
(5) Levins R. Evolution in changing environments: some theoretical explorations. *Princeton University Press* 1968.
(6) Gubry-Rangin C, Aigle A, Herrera-Alsina L. et al. Niche breadth specialization impacts ecological and evolutionary adaptation following environmental change. *ISME J* 2024;**18**:wrae183. https://doi.org/10.1093/ismejo/wrae183
(7) Peng ZH, van der Heijden MGA, Liu Y. et al. Agricultural subsoil microbiomes and functions exhibit lower resistance to global change than topsoils in Chinese agroecosystems. *Nat Food* 2025;**6**:375–88. https://doi.org/10.1038/s43016-024-01106-7
(8) Shenhav L, Thompson M, Joseph TA. et al. FEAST: fast expectation-maximization for microbial source tracking. *Nat Methods* 2019;**16**:627–32. https://doi.org/10.1038/s41592-019-0431-x
(9) CLSI. Performance standards for antimicrobial susceptibility testing: CLSI M100. *Clinical and Laboratory Standards Institute* 2026.
(10) Choi KH, Gaynor JB, White KG. et al. A Tn7-based broad-range bacterial cloning and expression system. *Nat Methods* 2005;**2**:443–8. https://doi.org/10.1038/NMETH765
(11) Chen M, Zhang WQ, Han LL. et al. A CysB regulator positively regulates cysteine synthesis, expression of type III secretion system genes, and pathogenicity in *Ralstonia solanacearum*. *Mol Plant Pathol* 2022;**23**:679–92. https://doi.org/10.1111/mpp.13189
(12) Herpell JB, Alickovic A, Diallo B. et al. Phyllosphere symbiont promotes plant growth through ACC deaminase production. *ISME J* 2023;**17**:1267–77. https://doi.org/10.1038/s41396-023-01428-7
(13) Zhang HP, Song JJ, Zheng ZR. et al. Fungicide exposure accelerated horizontal transfer of antibiotic resistance genes via plasmid-mediated conjugation. *Water Res* 2023;**233**:119789. https://doi.org/10.1016/j.watres.2023.119789
(14) Chen ZY, Lu J, Gao SH. et al. Silver nanoparticles stimulate the proliferation of sulfate reducing bacterium *Desulfovibrio vulgaris*. *Water Res* 2018;**129**:163–71. https://doi.org/10.1016/j.watres.2017.11.021
(15) Wu J, Zhou JH, Liu DF. et al. Phthalates promote dissemination of antibiotic resistance genes: an overlooked environmental risk. *Environ Sci Technol* 2023;**57**:6876–87. https://doi.org/10.1021/acs.est.2c09491
(16) Kim D, Paggi JM, Park C. et al. Graph-based genome alignment and genotyping with HISAT2 and HISAT-genotype. *Nat Biotechnol* 2019;**37**:907–15. https://doi.org/10.1038/s41587-019-0201-4
(17) Xie C, Mao XZ, Huang JJ. et al. KOBAS 2.0: a web server for annotation and identification of enriched pathways and diseases. *Nucleic Acids Res* 2011;**39**:W316–22. https://doi.org/10.1093/nar/gkr483
(18) Wu JD, Liu SJ, Zhang HY. et al. Flavones enrich rhizosphere *Pseudomonas* to enhance nitrogen utilization and secondary root growth in *Populus*. *Nat Commun* 2025;**16**:1461. https://doi.org/10.1038/s41467-025-56226-w
(19) Fraga CG, Clowers BH, Moore RJ. et al. Signature-discovery approach for sample matching of a nerve-agent precursor using liquid chromatography-mass spectrometry, XCMS, and chemometrics. *Anal Chem* 2010;**82**:4165–73. https://doi.org/10.1021/ac1003568
